# The TargetMine Data Warehouse: Enhancement and Updates

**DOI:** 10.3389/fgene.2019.00934

**Published:** 2019-10-09

**Authors:** Yi-An Chen, Lokesh P. Tripathi, Takeshi Fujiwara, Tatsuya Kameyama, Mari N. Itoh, Kenji Mizuguchi

**Affiliations:** Laboratory of Bioinformatics, National Institutes of Biomedical Innovation, Health and Nutrition, Osaka, Japan

**Keywords:** data warehouse, integrative data analysis, multi-omics data analysis, gene prioritisation, drug discovery, data mining, knowledge discovery

## Abstract

Biological data analysis is the key to new discoveries in disease biology and drug discovery. The rapid proliferation of high-throughput ‘omics’ data has necessitated a need for tools and platforms that allow the researchers to combine and analyse different types of biological data and obtain biologically relevant knowledge. We had previously developed TargetMine, an integrative data analysis platform for target prioritisation and broad-based biological knowledge discovery. Here, we describe the newly modelled biological data types and the enhanced visual and analytical features of TargetMine. These enhancements have included: an enhanced coverage of gene–gene relations, small molecule metabolite to pathway mappings, an improved literature survey feature, and *in silico* prediction of gene functional associations such as protein–protein interactions and global gene co-expression. We have also described two usage examples on trans-omics data analysis and extraction of gene-disease associations using MeSH term descriptors. These examples have demonstrated how the newer enhancements in TargetMine have contributed to a more expansive coverage of the biological data space and can help interpret genotype–phenotype relations. TargetMine with its auxiliary toolkit is available at https://targetmine.mizuguchilab.org. The TargetMine source code is available at https://github.com/chenyian-nibio/targetmine-gradle.

## Introduction

The rapid proliferation of high-throughput omics technologies has revolutionised biological research by significantly adding new omics data. However, as the experimental datasets increase in size and complexity, extraction of meaningful biological knowledge becomes qualitatively more difficult, expensive and labourious. Therefore, there is an ever widening gulf between data generation and the rate at which it can be properly analysed (Greene et al., 2014). Proper mining and curation of large biological datasets are necessary to develop an improved understanding of living systems and of disease pathogenesis.

An integrative multi-omics approach combines different types of biological data into a single analytical framework to understand the relationships between different cellular components (Zhu et al., 2012; Yan et al., 2018). Such analyses are useful to develop analytical models that can interpret genotype–phenotype relationships, garner the knowledge of pathways involved in cellular events and diseases, help pinpoint targets (such as gene and proteins) of biological and therapeutic interest and potentially develop intervention methods than can counteract undesirable phenotypic progression (i.e. diseases) (Sun and Hu, 2016; Hasin et al., 2017).

A major challenge in multi-omics data analysis is the availability of clean and usable biological data. We have previously developed TargetMine, an integrated data warehouse based on the object-oriented InterMine data warehouse framework (Smith et al., 2012; Kalderimis et al., 2014; Triplet and Butler, 2014), which models biological entities (such as genes and proteins) as ‘objects’ that are described by a set of attributes and their relationships with other objects are modelled as ‘references’. The InterMine system allows for integration of different types of biological databases, and it comes pre-equipped with data integration features that are able to directly parse the data from commonly used data formats and sources (such as UniProt, OBO, FASTA and BioPAX). InterMine also allows the users to design their own data parsers (Smith et al., 2012; Lyne et al., 2007; Kalderimis et al., 2014). The TargetMine data model was developed by extending a customised version of the core InterMine data model. When integrating similar types of data from heterogeneous data sources, we first identified common attributes (gene identifiers for instance) which are then used to merge the overlapping datasets into a suitable data model. The data sources are prioritised based on their reliability, and the stored identifiers are constantly revised to update or discard outdated identifiers with every database update (Lyne et al., 2007; Smith et al., 2012).

TargetMine was initially developed and optimised for target discovery and prioritisation of candidate genes, especially in early stage drug discovery (Chen et al., 2011). We have continued to make significant additions and refinements to the TargetMine system to transform TargetMine into an integrative data analysis platform that can more effectively interpret information-rich omics datasets for biological knowledge discovery (Chen et al., 2019). Aside from periodically updating the existing datasets, these new developments have involved assimilation of newer biological data types and a new auxiliary toolkit to assist with data analysis and visualisation that addresses the limitations in the core InterMine framework (Chen et al., 2016). 

Here, we describe our progressive efforts to enhance TargetMine as a data analysis platform that can better assist multi-omics data analysis and biological knowledge discovery especially in disease biology. These efforts broadly fall into three categories: (1) upgrading the existing data types with the up-to-date information available from the source repositories; (2) assimilating new data types, especially those data types that help to examine different types of gene-gene relations; and (3) augmenting the auxiliary toolkit to better analyse and visualise biological data.

We will now describe our efforts below individually.

## Additional Data Sources and Data Models in Targetmine Provide a Deeper Coverage of the Biological Data Space

A comprehensive coverage of the biological data space is necessary for drug discovery and related research. To achieve this, we have continuously expanded the repertoire of data types in TargetMine. Since the last major release, we have included within TargetMine new biological data associated with three major areas—drug-target interactions, gene-disease associations and biological mechanisms. The inclusions of these data types have offered deeper insights into gene-gene relations and have also enabled the users to perform more probing biological queries with TargetMine (**Table 1**). To enable the user to quickly and easily perform complex queries, TargetMine contains a library of ‘templates’ that consist of predefined queries with a simple form and description and are categorised by data types (Chen et al., 2011; Chen et al., 2016; Chen et al., 2019).

**Table 1:** Key enhancements and updates in TargetMine since the last published iteration (2016).

Data types, data models and features	New and/or enhanced data types and features	Existing data types and features
**Protein–protein interactions**	KEGG relations; Post-translation modifications (phosphorylation); PSOPIA-likelihood scores for all PPIs; Gene co-expression scores for HCDPs (GCE-HCDP)	Combined PPI repository from iRefindex and BioGRID, literature; Classification of PPIs as HCs and HCDPs
**Metabolomics**	KEGG COMPOUND –pathway mapping; KEGG reactions	KEGG COMPOUND
**Gene-disease relations**	ClinVar variations; dbSNP publications; DisGeNET associations	GWAS data from NHGRI
**Literature mining**	MeSH term descriptors; Publication abstracts	NCBI PubMed links
**TF-target interactions**	∼400,000 human and mouse TF-target annotation from ENCODE	Amadeus; ORegAnno; HTRIdb
**TargetMine auxiliary toolkit**		
**Composite interaction network**	Filter PPIs by HCDPs; Filter interaction types by expressed-tissues and GEL; Add directed PPIs from KEGG; Restrict interaction types to within the user-supplied gene list	Include multiple interaction types
**Association heatmap**	Dendrogram of hierarchically assembled associations with distances; Expressed-tissue feature	Two-colour grid of squares

### KEGG Relations

KEGG (Kyoto Encyclopedia of Genes and Genomes) is a collective repository of genes, genomes, pathways, diseases and chemical compounds that provides a comprehensive mapping of the biological systems (Kanehisa et al., 2016). The relation element in KEGG typically specifies relationship between two entities (proteins and compounds) in KEGG pathways. KEGG relations largely correspond to signalling pathway maps and encode regulatory information such as ‘A activates B’, ‘A inhibits B’ and ‘A phosphorylates B’. The inclusion of KEGG relations is useful, since they often provide an additional context to interactions between gene products that are not always evident from standard PPI analysis. This integration has enabled the users to reconstruct probable signal transduction paths by performing queries such as ‘Given a pair of genes A and B, find an intermediate gene and relations from gene A to gene B’.^1^

### Post-Translational Modifications

Post-translational modifications (PTMs) are events that involve covalent addition of functional groups to proteins or their proteolytic processing during and after their biosynthesis. PTMs amplify the functional diversity of proteins and expand their influence over various cellular processes. Therefore, identifying and understanding PTMs help in a deeper understanding of cellular functions and in disease biology (Mnatsakanyan et al., 2018; Thygesen et al., 2018). We retrieved PTM associations from PhosphoSitePlus (Hornbeck et al., 2015), a knowledgebase of mammalian PTMs, and we carefully parsed the UniProt sequence annotation (features) that describe regions or sites of interest in proteins, to create an integrated repository of PTMs in TargetMine. The inclusion of PTMs in TargetMine enables the users to perform complex queries such as ‘Given a list of proteins, identify upstream kinases that may phosphorylate them’^2^ or ‘Given a protein and a specific residue position, identify any PTMs mapped to that residue’.^3^

### KEGG Reactions Compound-Pathway Mappings

Metabolites are the low molecular weight compounds such as amino acids, sugars and lipids, which are typically substrates and by-products of biological processes and enzymatic reactions; they are widely involved in feedback regulatory processes in the cell and, being the downstream products, often directly influence the phenotype. Thus, the metabolome is often regarded as the link between genotype and phenotype (Krumsiek et al., 2016). To facilitate a more effective metabolomics analysis with TargetMine, we first extended the existing compound class to create a new KEGG COMPOUND class. Subsequently, we referenced the KEGG COMPOUND class both with the existing Enzyme and Pathway classes using the relationships extracted from the KEGG COMPOUND database. We also defined a new Reaction class to describe the biochemical reactions in the KEGG reaction database, and this class was referenced with all of the KEGG COMPOUND, pathway and enzyme classes. Given a list of compounds, the users can now retrieve the corresponding enzymes involved in their metabolism, the enzymatic reactions involving these metabolites and, even map them to the corresponding pathways and diseases associated with the pathways. Users can also perform enrichment analyses to prioritise the enzymes/genes and pathways specifically associated with their metabolites of interest (see example below).

### Disease-Gene Mappings

A deeper understanding of disease pathogenesis requires a mapping of links between genes, pathways and specific diseases, but they are difficult to obtain in general. Recently, we have enhanced the integration of genetic linkages to diseases by improving the existing GWAS data model and adding the variation annotations from ClinVar (Landrum et al., 2015), and the disease associations that were extracted from the associated publications in dbSNP (Chen et al., 2019). We have also included gene-disease associations compiled in DisGeNET, an integrative platform of curated gene-disease associations (Pinero et al., 2017). This integration has enabled the users to perform queries such as ‘Given a gene, find the related SNPs and any diseases associated with these SNPs’.^4^

### Scientific Literature Survey

Literature survey is indispensable to annotating gene information, interpreting gene sets and facilitating further research. However, scientific literature is increasing exponentially, making it difficult for the researchers to find, study and understand new publications of interest. To facilitate an easier sharing of scientific knowledge, we have incorporated document representations such as MeSH (Medical Subject Headings) (Rogers, 1963) descriptors (such as general article, review, clinical study, case report, etc.) and abstracts into TargetMine. This implementation allows the users to quickly screen for scientific texts (based on their MeSH descriptors) associated with their gene(s) of interest. For example, users may restrict their query to retrieving only those publications classified as ‘case report’ by constraining the ‘Mesh Terms’-> ‘Identifier’ attribute for the MeSH Terms identifier ‘D002363’ (case reports). Researchers typically rely on abstracts to assess an article for further reading and often; abstracts are the only source of information that are freely available (Germini et al., 2017). To allow the users to easily access and scan article abstracts of interest, we leveraged the attribute ‘Abstract Text’ within the ‘Publication’ class. This implementation allows the users to retrieve publications associated with their gene(s) of interest along with their corresponding abstracts; this implementation also allows the users to quickly and easily scan multiple abstracts in a single webpage, instead of visiting the individual ‘Publication report’ pages and clicking on the available PMID links to access the corresponding abstracts on NCBI PubMed (as was the case previously).

## Inclusion of Computationally Predicted Associations and Scores in Targetmine

### TF-Target Associations

Transcription factor (TF)–target gene interactions determine gene expression patterns, and therefore, regulate cellular functions. Previously, we had included expert-curated experimentally validated human TF-target gene interaction data from Amadeus (Linhart et al., 2008), ORegAnno (Griffith et al., 2008) and HTRIdb (Bovolenta et al., 2012) to create a combined repository in TargetMine (Chen et al., 2016). To expand the coverage of TF-target gene interactions, we examined and processed the vast amounts of TF-binding site data compiled by the Encyclopedia of DNA Elements (ENCODE) consortium (see *Methods*). Over 200,000 new TF-target gene interactions corresponding to 23 human TFs were incorporated into TargetMine in this manner. We also incorporated nearly 200,000 TF-target gene interactions corresponding to 39 TFs in the mouse genome, thereby providing a detailed coverage of gene-regulatory associations in mouse that were not available in the previous iterations of TargetMine.

### PPI Confidence Scores Using Predicted Likelihood of PPIs

PPIs are vital to virtually every cellular process, and their dysregulation typically leads to cellular dysfunction including diseases. However, it is necessary to assess the PPI data properly to ensure the robustness of PPIN-based analyses in investigating disease-causing biological pathways and to discover druggable target proteins. We had previously performed a confidence assessment of our combined PPI repository and defined a reliable high-quality subset termed ‘high-confidence direct physical PPIs’ (HCDPs) (Chen et al., 2016). HCDPs have been helpful in analysing network topological properties and identifying key components of the presently characterised interactome maps, namely, network ‘hubs’ and ‘bottlenecks’ (Tripathi et al., 2013; Chen et al., 2016). However, HCDPs constitute only a small proportion of all available PPIs, and using HCDPs alone for PPI-based network analysis may often exclude potentially useful PPIs. This is especially true for the mouse and rat interactomes in TargetMine, where HCDPs are rather sparse. Therefore, we have included an additional measure for the assessment of PPI reliability. In our group, we had previously developed prediction server of protein–protein interactions (PSOPIA), an integrative averaged one-dependence estimators (AODE)–based method to predict the likelihood of interaction between a pair of proteins based on experimentally characterised homologous PPIs (Murakami and Mizuguchi, 2014). We employed PSOPIA to evaluate all PPIs within TargetMine, and the output PSOPIA scores were tagged to the individual PPIs as a new attribute PsopiaScore. This implementation has enabled the users to query the interacting partners of a gene/protein or a list of genes/proteins of interest and to infer overall PPI networks involving these genes/proteins consisting of all interactions judged to be of sufficiently high quality by the user, either based on their HCDP status and/or their PSOPIA scores.

### Gene Co-Expression Analysis for Prediction of Novel DNA-Binding Proteins and for Improved PPI-Based Network Analysis

Gene expression refers to the process where the genetic information encoded in a gene is transcribed into a functional gene product—RNA or the eventual protein. Gene expression analysis involves mapping and analysing collective gene expression patterns that dictate cellular function under different environments. The spread of technologies that can map global gene expression profiles has led to an abundance of genome-wide gene expression data (transcriptome) for many cell and tissue types and physiological conditions (Barrett et al., 2012; Kolesnikov et al., 2015). Global gene expression profiles have been variously analysed to search for genes that are differentially expressed in different cellular and physiological conditions (such as development, infection and diseases) and to predict functions for genes of unknown function. A guiding principle of function prediction using gene expression is *guilt-by-association*, which assumes that genes with related functions are more likely to have correlated properties such as interactions and expression patterns (Singer et al., 2004).

**Figure 1:**
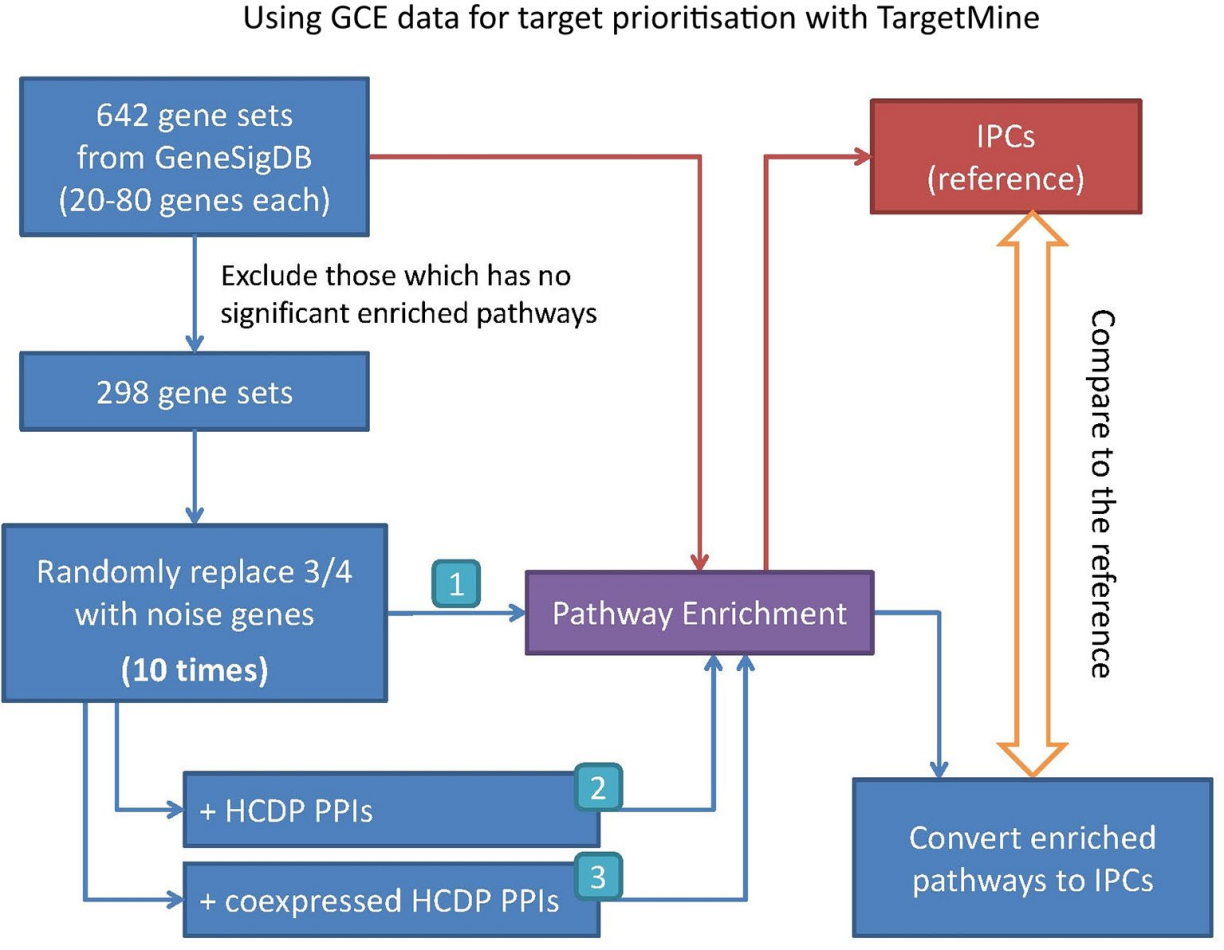
Assessing the potential benefits of including GCE data in target prioritisation with TargetMine. GCE, gene co-expression; HCDP, high-quality direct physical PPIs; IPC, integrated pathway clusters; PPI, protein–protein interactions.

**Figure 2:**
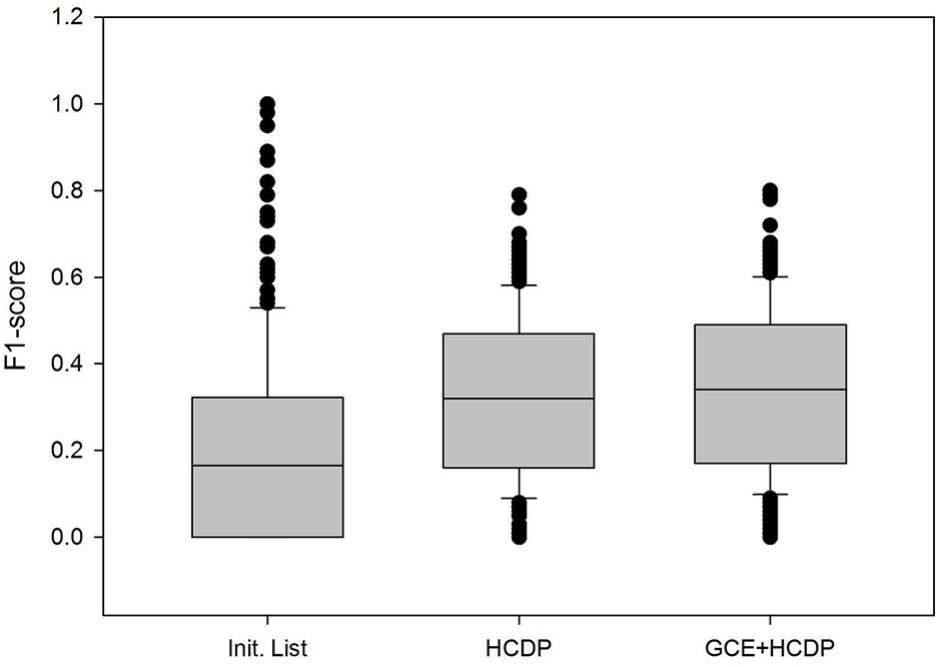
The inclusion of HCDPs filtered by Gene co-expression (GCE-HCDP) to generate extended gene sets led to an overall improved target prioritisation performance when compared with the inclusion of unfiltered HCDPs and un-extended gene sets.

In this study, we have sought to leverage global gene co-expression (GCE) patterns to minimise biological noise and to further refine and improve PPI-based network analysis (**Figure 1**). To assess the effectiveness of this approach, we performed GSFE analysis on a multiple gene sets gathered from literature and then repeated the tests on modified gene sets that included both co-expressed genes and randomly selected unrelated genes (biological noise) (see methods). Among the 298 curated gene sets that were tested, 81% (240) were associated with overall higher F1 scores when HCDPs or globally co-expressed HCDPs (GCE-HCDP) where added to the initial gene list, compared with initial gene sets (**Figure 2**; **Table 2**, **S1**). Furthermore, of the 240 gene sets where GCE showed an improved prioritisation performance, in 170 of them (∼70%), inclusion of GCE-HCDP contributed to a better performance in terms of F1 scores as compared to inclusion of HCDPs alone (**Figure 2**; **Table 2**).

**Table 2:** The inclusion of HCDPs filtered by gene co-expression (GCE-HCDP) to generate extended gene sets led to an overall improved target prioritisation performance when compared with the inclusion of unfiltered HCDPs and un-extended gene sets.

**a. Average F1-score**		
Original test	0.211	
+HCDP	0.327	
+Co-exp-HCDP	0.341	
**b. T-test**		
	**Original test**	**+HCDP**
+HCDP	5.77×10^−18^	
+Co-exp-HCDP	5.93×10^−22^	6.14×10^−20^

Our observations suggested that the inclusion of HCDPs and GCE-HCDPs contributed to improved target prioritisation and gene set analysis with TargetMine.

## Enhanced Data Analysis and Visualisation Workflow With Targetmine

We had previously developed an auxiliary toolkit to assist with data analysis and visualisation in TargetMine without any scripting and/or programming efforts on the part of the user (Chen et al., 2016) (https://targetmine.mizuguchilab.org/tutorials/auxiliary-toolkit/). We have subsequently added new analytical and visualisation features to further enhance the ability of TargetMine as a data analysis platform. For instance, we have now added a dendrogram to the association heatmap function, which permits users to quickly and more easily identify clusters of genes that share significant functional attributes. We have also introduced the ‘Expressed Tissue’ feature that allows the users to hierarchically assemble a heatmap of user-supplied genes and the cell/tissues where they are highly expressed and thereby obtain a contextual view of their expression patterns. We have also enhanced the efficacy of the network visualisation function. In the present form, the function would permit the users to supply a list of genes and construct and visualise a composite interaction network that includes all the biomolecular interactions within TargetMine, i.e. PPIs, MTIs, PCIs/drug-target interactions and TF-target gene interactions that are associated with the query genes. However, adding too many interactions can also render the network very dense and complex, therefore becoming difficult to load and visualise properly in the browser. To address these concerns, we have added a series of features to select and filter biomolecular interactions by qualitative assessment and/or contextual information. For instance, the ‘Interaction Network’ feature allows the user to restrict the PPI selection to ‘HCDPs’ or expand them to include ‘All’ PPI types by selecting the corresponding circles. We have also introduced features that permit the users to filter the interaction types by expressed tissues and GELs. We have also introduced a feature to allow the users to specifically include and visualise directed gene–gene relations parsed from KEGG. Moreover, we have improved the network feature to restrict the MTIs, PCIs/drug-target interactions and TF-target gene interactions to the user supplied gene list.

## Applications With Use Cases

### Trans-Omics Data Analysis

To demonstrate the effectiveness of TargetMine in assisting multi-omics data analysis, we re-examined a previously published multi-omics data on mitochondrial links to liver metabolism, and the effects of a high-fat diet on it (Williams et al., 2016). We first retrieved the biomolecules (110 transcripts, 27 proteins and 25 metabolites) that were differentially expressed in high-fat diet (HFD) fed mice relative to control (see methods). Next, the differentially expressed transcript, protein and metabolite sets were transformed into the corresponding transcriptome, proteome and metabolome differentially expressed gene (DEG) sets, respectively (see **Supplementary File S1** for the detailed methodology with the help of an example). The inferred DEG sets (containing 84, 29 and 62 genes, respectively; **Supplementary Table S1**, **Supplementary Figure S1**) were first compared (using the list operations in TargetMine) to identify overlapping genes. Three DEGs (Ces2a, Cyp3a11 and Csad) were shared across transcriptome and proteome DEG sets, and a solitary gene, cysteine sulfinic acid decarboxylase (Csad), was downregulated across all the three DEG sets (**Supplementary File S1B**). Csad is an enzyme that plays a key role in generating taurine from cysteinesulfinate in liver, and its hepatic expression and abundance are typically downregulated by bile acids responsible for modulating lipid metabolism (Kerr et al., 2014). Our observations, therefore, clearly suggested that the fluctuations in Csad levels were likely to be modulated by dietary fat *via* bile acids. Additionally, we were also able to prioritise signatures that were consistent with an HFD model such as cytochrome p450 subunit Cyp3a11 that functions in retinoic acid metabolism.

Next, the individual DEG sets were then subjected to pathway enrichment analysis (see methods). Five enriched pathways were associated with the transcriptome DEG set, 11 enriched pathways with the proteome DEG set, and 17 enriched pathways were associated with the metabolome DEG set, respectively. KEGG pathway sub-categories ‘Lipid metabolism’, ‘Global and overview maps’, ‘Cancers: Overview’ and ‘Metabolism of cofactors and vitamins’ were commonly represented in the enriched pathways across all the three DEG sets (**Supplementary File S1B**). Specifically, KEGG pathway ‘Metabolic pathways’ was commonly enriched in all the three DEG sets; ‘Steroid hormone biosynthesis’, ‘Linoleic acid metabolism’, ‘Retinol metabolism’, ‘Arachidonic acid metabolism’ and ‘Chemical carcinogenesis’ were commonly enriched in Transcriptome and Proteome DEG sets (associated with Cyp3a11), and ‘Drug metabolism—other enzymes’ was commonly enriched in Proteome and Metabolome DEG sets, respectively (although no gene overlap was observed). Taken together, our observations have suggested that higher levels of dietary fat are responsible for dysregulation of cellular factors and pathways associated with lipid metabolism and as such have provided promising candidates for further research.

### Extracting Gene-Disease Associations From Literature Using Mesh Descriptors

A vast amount of untapped associations between genes and diseases are scattered across biomedical literature. A quick and efficient mining of such information can help interpret genotype-phenotype relationships and also speed up database curation. The inclusion of MeSH descriptors now allows the TargetMine users to easily survey for annotated gene-disease associations for their gene(s) of interest. As a case study, we extracted literature-embedded gene-disease associations for PPARγ (peroxisome proliferator–activated receptor gamma), a nuclear receptor that is implicated in the pathology of numerous diseases including obesity, diabetes and cancer. Next, we sought to retrieve all the publications that were indicative of the involvement of PPARγ in disease pathogenesis by constraining the query for MeSH term attribute ‘Diseases’. We retrieved 397 unique disease associations for PPARG in this manner (**Supplementary File S2**).

## Conclusions

TargetMine is a versatile data analysis platform that provides a unified, homogenous representation of diverse types of omics and other biological data; it allows the users to query and navigate across the stored data types and analyse them in a singular interface. In this study, we have described the augmentation of the TargetMine by progressively improving and expanding the coverage of data types and by adding new and improved analytical features. We have also demonstrated how the extension of TargetMine system has significantly boosted its capabilities to survey the biological target space, to assist multi-omics data analysis, to interpret novel genotype-phenotype relationships and to facilitate biologically relevant knowledge discovery, especially in disease biology.

For the future developments, we will continue to accommodate new and emerging data types of interest and expand the analytical features. We also aim to introduce a workflow function for multi-omics data analysis that will allow the users to more easily and effectively analyse and interpret their omics datasets and advance their research.

## Methods

### Tf-Target Gene Associations From Encode

The binding events (peaks) for human and mouse TFs with binding profiles in different cell types were downloaded from the ENCODE resource (Davis et al., 2017). To accommodate the additional TF-target information, we redefined the erstwhile protein-DNA interaction class into a new transcriptional regulation class. The promoter region was defined as 10,000-bp upstream of the transcriptional start site (TSS). We extracted binding site positions within this hypothesised promoter region, and we identified the corresponding genes by mapping the genomic coordinates downstream of the TSS to the genomic coordinates stored within TargetMine. Next, we mapped the TFs whose binding sites were identified in this manner with the downstream genes to generate new TF-target gene associations.

### Gene Co-Expression Analysis

#### Data Sets

The gene sets were retrieved from GeneSigDB, a database of curated gene signatures (Culhane et al., 2012). To obtain a reasonable size of candidates, we selected only the human, mouse and rat gene sets that consisted of 30∼80 genes; 642 gene sets were selected in this manner. The genes in the so-called ‘standardised’ gene list in GeneSigDB were represented by Ensembl identifier and symbol; for further analysis, we mapped them to Entrez Gene identifier (Gene ID) using TargetMine (build 20160629). The Ensembl identifiers that were not mapped to a corresponding Gene ID were excluded from the list, thereby marginally reducing the sizes of the gene sets. Next, we performed pathway enrichment analysis on each of these gene sets, and only those gene sets (298 out of 642) that were associated with at least one enriched pathway (pathways were judged to be significant if the adjusted *p*-value was 0.05 or less) were taken up for subsequent analyses (**Figure 1**).

#### Global Gene Co-Expression Analysis

Global gene expression profiles for human genes were retrieved from gene expression omnibus (GEO) (Barrett et al., 2012; Kolesnikov et al., 2015), and gene co-expression levels were computed as described earlier (Ahmad et al., 2018).

#### Gene Prioritisation With PPI and GCE

The aim of the gene prioritisation is to identify a relatively important subset of genes form a list of candidates for further analysis. The first step of target prioritisation within TargetMine involves uploading a list of initial candidate genes or proteins (e.g. a set of differentially expressed genes or a set of proteins that interact with a given protein) to create a TargetMine gene list. Next, the enrichment of specific biological themes such as KEGG/Reactome pathways, integrated pathway cluster (IPC) (Chen et al., 2014), gene ontology terms etc. is estimated by performing Fisher’s exact test here followed by multiple testing correction to control the false discovery rate. The genes associated with the most significantly enriched biological associations (that satisfied, in this instance, a condition of *p* ≤ 0.05 after a multiple test correction with the Benjamini and Hochberg procedure (Benjamini and Hochberg, 1995; Benjamini et al., 2001)) are judged to be highly important to the biological phenomenon under study and therefore selected for further analyses.

For each of 298 gene sets, we replaced at random 75% genes with an equal number of unrelated randomly selected genes from the corresponding genome to generate test gene sets to incorporate biological noise. To avoid any bias incurred due to the selection of random genes, the process was repeated 10 times to infer 10 test gene sets for each curated gene list. Next, the HCDPs for the genes within the test gene sets were retrieved from TargetMine and were appended to the initial test gene sets to create extended test gene sets. Independently, co-expressed HCDPs for the test gene sets were retrieved from TargetMine and were appended to the initial test gene sets to create extended test gene sets. Only the interacting partners that had a GCE value greater than 0.03 or less than −0.03 with the genes in the test gene sets were considered. Finally, the prioritisation tests (**Figure 1**) were then performed for each test gene set.

#### Evaluating the Performance of GCE-Filtered HCDPs in Target Prioritisation

To evaluate the protocols, we compared the enriched pathways among the reference gene sets, the ‘noisy’ gene sets and the extended noisy gene sets that were independently generated with the inclusion of HCDPs and GCE-HCDPs.

The enriched pathways (*p*-value < 0.05) were then mapped to their corresponding IPC (Chen et al., 2014). These enriched IPCs from the reference gene set were defined as the true positives (TPs) in this instance, and the rest were defined as false positives (FPs). TPs which were not found in the test results were defined as false negatives (FNs). For each gene set, the F1-score was estimated as follows: 

$$F1=\frac{2\mathrm{TP}}{2\mathrm{TP}+\mathrm{FP}+\mathrm{FN}}$$

The test was performed 10 times for each gene set (from the step that we randomly generated the ‘noisy’ gene set). A student t-test was also performed to compare the significance of the differences between the two approaches. If the new prioritisation protocol achieved an overall higher F1-score than standard enrichment analysis, it was assumed to have provided an improved prioritisation performance, even the difference was trivial.

#### Gene Set Inference and Pathway Enrichment for Multi-Omics Data Analysis

The biomolecules (transcripts/genes, proteins and metabolites) were judged to be differentially expressed if they were statistically significantly (*p* ≤ 0.05; t-test) increased or decreased more than 1.5-fold i.e. if the fold change (FC) ≥1.5 (upregulated) or FC ≤ 0.667 (downregulated) in mice fed with high-lipid diet relative to the control. The biological pathway data from KEGG were used to assign functional annotations to the DEGs, using TargetMine. Statistical significance of the pathway enrichment was determined by Fisher’s exact test, and the *p*-values were corrected for multiple testing using the Benjamini–Hochberg procedure. The enriched pathways were considered statistically significant if the adjusted *p* ≤ 0.05.

## Data Availability Statement

All datasets generated for this study are included in the manuscript/**Supplementary Files**.

## Author Contributions

Y-AC and LT contributed equally to this work. Y-AC, LT, TF, TK, MI, and KM were responsible for data gathering and validation. Y-AC, LT, TF and TK performed all the data analysis. Y-AC, LT, and KM were responsible for overall data analysis and interpretation and wrote the manuscript. All authors read and approved the final version of the manuscript.

## Funding

This work was in part supported by Grants-in-Aid for Scientific Research from the Japan Agency for Medical Research and Development (Grant Number 19ak0101068h0003; “The adjuvant database project” Grant Number 16ak0101010h0005) and from the Japan Society for the Promotion of Science (Grant Number 17K07268) to KM.

## Conflict of Interest

The authors declare that the research was conducted in the absence of any commercial or financial relationships that could be construed as a potential conflict of interest. 

The handling editor declared a past co-authorship with the authors.
