# Supplementary material for: The TargetMine Data Warehouse: Enhancement and Updates
Source: Front Genet. 2019 Oct 9;10:934. doi: 10.3389/fgene.2019.00934 (PMC6794636; doi:10.3389/fgene.2019.00934)
Supplement: Supplementary file 2 [file DataSheet_1.pdf]

Supplementary File S1: Trans-omics data analysis with TargetMine-Transforming metabolomics data into other omics data types.

In this example, we demonstrate how to process metabolomics datasets to map the associated metabolic reactions, enzyme activities and the context-specific (such as specific cell/tissue-type) expression of the enzyme-coding gene and to facilitate integrative trans-omics analysis with TargetMine. Here we use “pyruvate” as an example.

Note: The users can click the “+” symbol to expand the class/node of interest and view the available nodes/classes within.

Step 1: Use the keyword search in TargetMine home page and obtain the list of entries associated with the term “pyruvate”. For further analyses, we retrieved the KEGG Compound ID for “pyruvate” that is “C00022”.

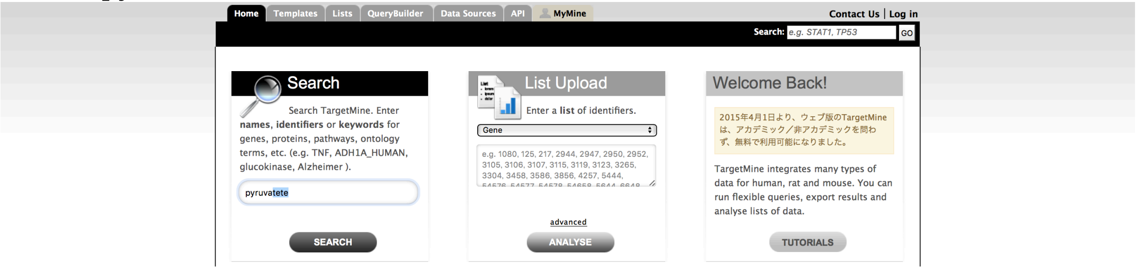

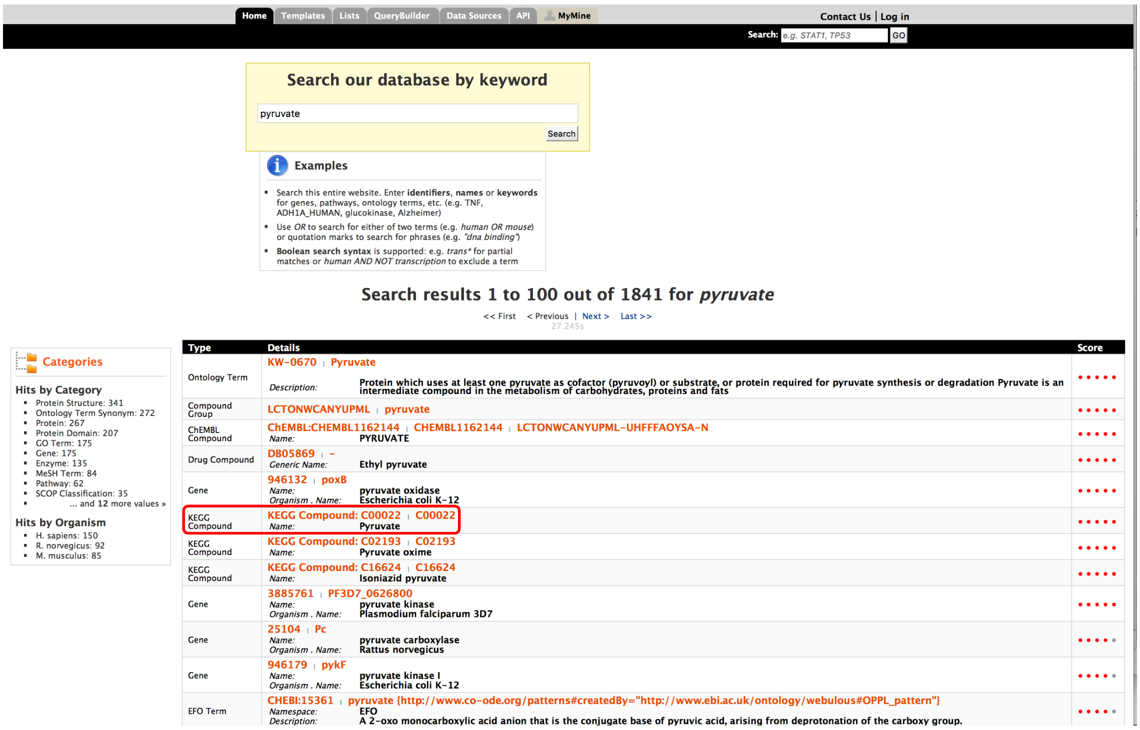

Step 2: Click the “QueryBuilder” tab in the tab panel

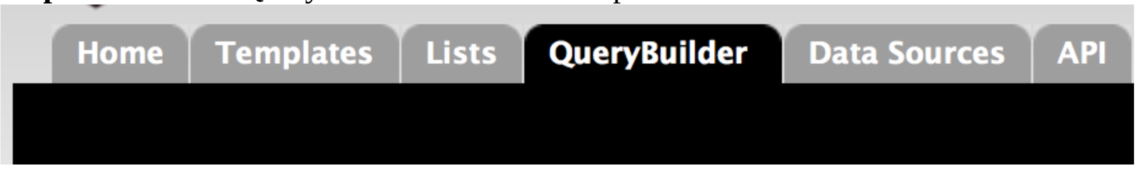

Step 3: Select “KEGG Compound” from the drop-down menu of data types and click the “Select” button.

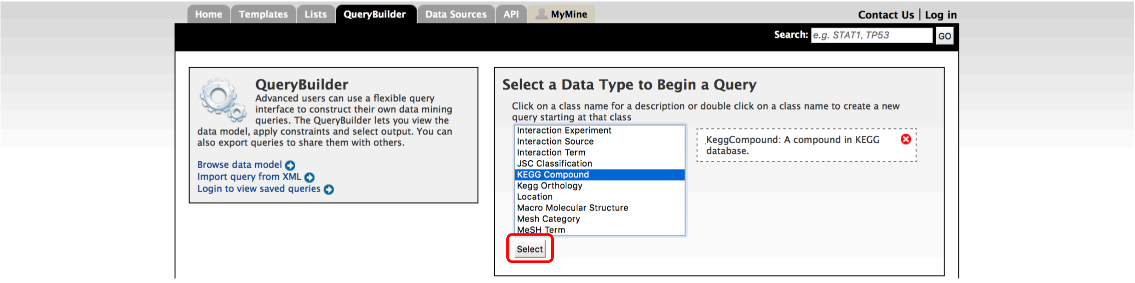

Step 4-A: In the model browser, select “KEGG Compound” and “KEGG Compound > Enzymes” and then and click the corresponding labels (KEGG Compound - “InChIKey”, “Name”, Original Id”; Enzyme- “Description”, “EC Number”) to select the desired attributes for output.

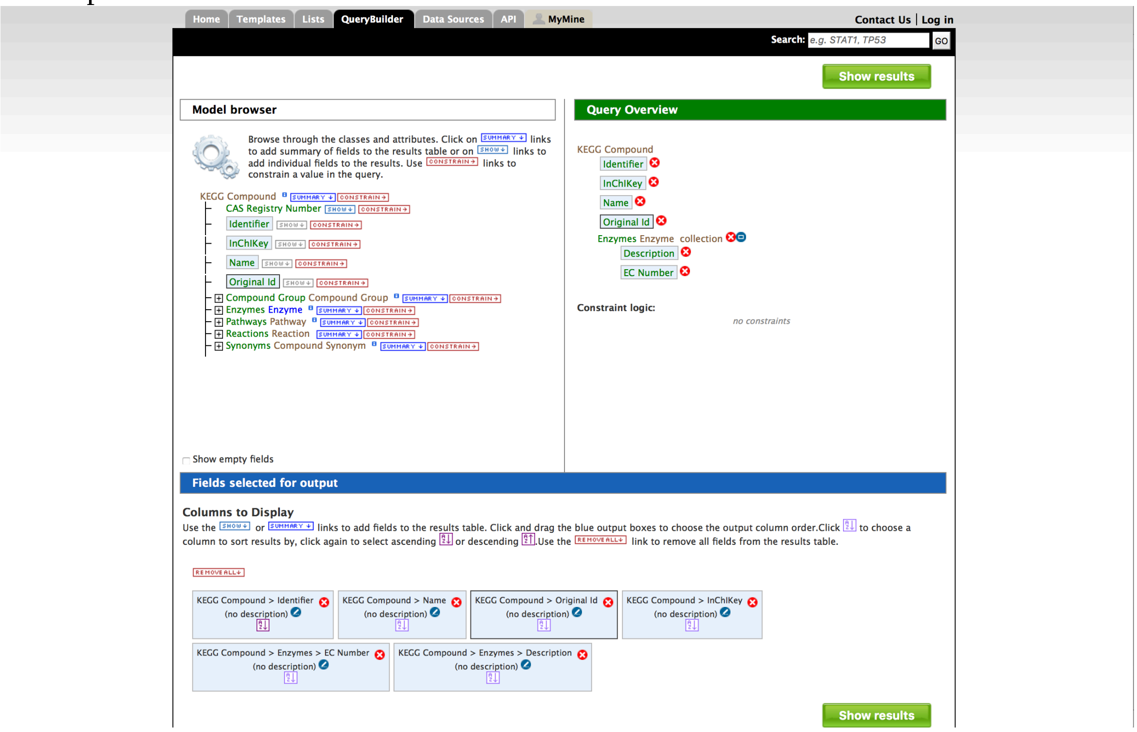

Step 4-B: To restrict the output results, we have constrained the query for C00022 (pyruvate) using the “CONSTRAIN” function.

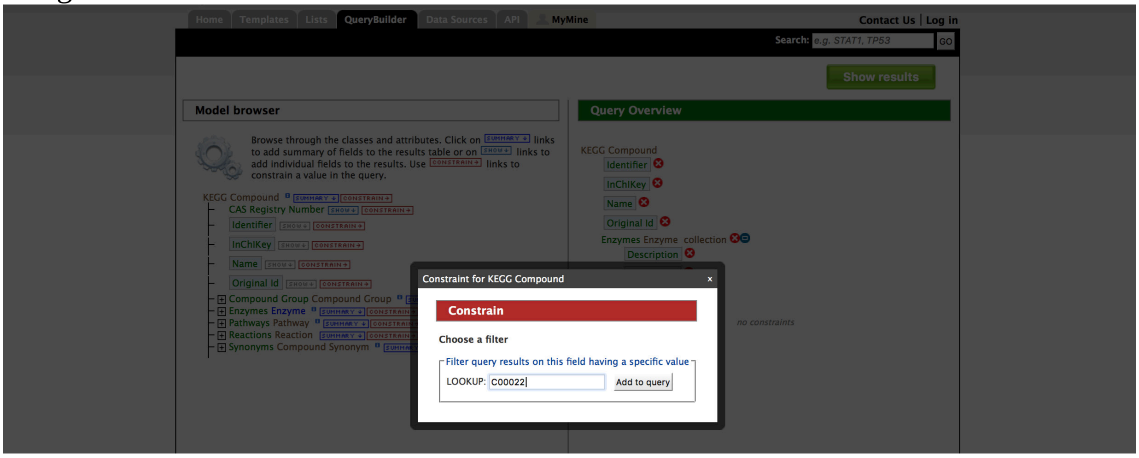

Step 4-C: Click "Show results" button to display the output.

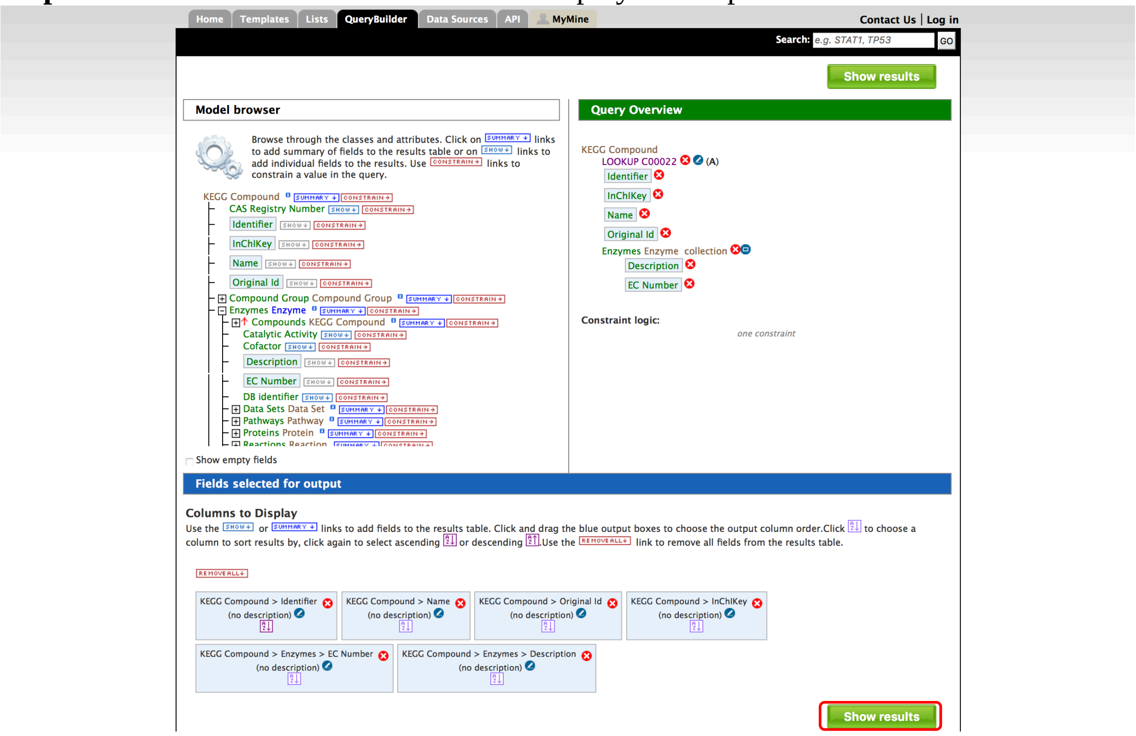

Step 4-D: The output table returns a list of Enzymes that are associated with KEGG reactions involving pyruvate.

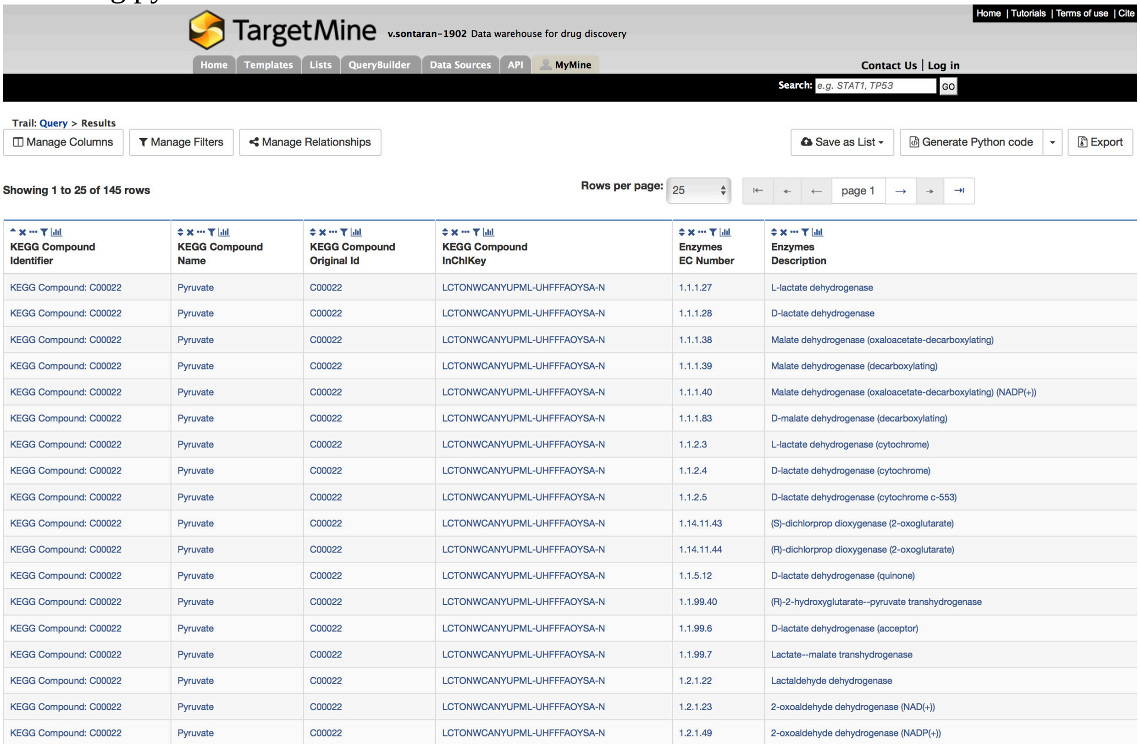

KEGG Compound Identifier	KEGG Compound Name	KEGG Compound Original Id	KEGG Compound InChIKey	Enzymes EC Number	Enzymes Description
KEGG Compound: C00022	Pyruvate	C00022	LCTONWCANYUPML-UHFFFAOYSA-N	1.1.1.27	L-lactate dehydrogenase
KEGG Compound: C00022	Pyruvate	C00022	LCTONWCANYUPML-UHFFFAOYSA-N	1.1.1.28	D-lactate dehydrogenase
KEGG Compound: C00022	Pyruvate	C00022	LCTONWCANYUPML-UHFFFAOYSA-N	1.1.1.38	Malate dehydrogenase (oxaloacetate-decarboxylating)
KEGG Compound: C00022	Pyruvate	C00022	LCTONWCANYUPML-UHFFFAOYSA-N	1.1.1.39	Malate dehydrogenase (decarboxylating)
KEGG Compound: C00022	Pyruvate	C00022	LCTONWCANYUPML-UHFFFAOYSA-N	1.1.1.40	Malate dehydrogenase (oxaloacetate-decarboxylating) (NADP(+))
KEGG Compound: C00022	Pyruvate	C00022	LCTONWCANYUPML-UHFFFAOYSA-N	1.1.1.83	D-malate dehydrogenase (decarboxylating)
KEGG Compound: C00022	Pyruvate	C00022	LCTONWCANYUPML-UHFFFAOYSA-N	1.1.2.3	L-lactate dehydrogenase (cytochrome)
KEGG Compound: C00022	Pyruvate	C00022	LCTONWCANYUPML-UHFFFAOYSA-N	1.1.2.4	D-lactate dehydrogenase (cytochrome)
KEGG Compound: C00022	Pyruvate	C00022	LCTONWCANYUPML-UHFFFAOYSA-N	1.1.2.5	D-lactate dehydrogenase (cytochrome c-553)
KEGG Compound: C00022	Pyruvate	C00022	LCTONWCANYUPML-UHFFFAOYSA-N	1.14.11.43	(S)-dichlorprop dioxygenase (2-oxoglutarate)
KEGG Compound: C00022	Pyruvate	C00022	LCTONWCANYUPML-UHFFFAOYSA-N	1.14.11.44	(R)-dichlorprop dioxygenase (2-oxoglutarate)
KEGG Compound: C00022	Pyruvate	C00022	LCTONWCANYUPML-UHFFFAOYSA-N	1.1.5.12	D-lactate dehydrogenase (quinone)
KEGG Compound: C00022	Pyruvate	C00022	LCTONWCANYUPML-UHFFFAOYSA-N	1.1.99.40	(R)-2-hydroxyglutarate--pyruvate transhydrogenase
KEGG Compound: C00022	Pyruvate	C00022	LCTONWCANYUPML-UHFFFAOYSA-N	1.1.99.6	D-lactate dehydrogenase (acceptor)
KEGG Compound: C00022	Pyruvate	C00022	LCTONWCANYUPML-UHFFFAOYSA-N	1.1.99.7	Lactate--malate transhydrogenase
KEGG Compound: C00022	Pyruvate	C00022	LCTONWCANYUPML-UHFFFAOYSA-N	1.2.1.22	Lactaldehyde dehydrogenase
KEGG Compound: C00022	Pyruvate	C00022	LCTONWCANYUPML-UHFFFAOYSA-N	1.2.1.23	2-oxoaldehyde dehydrogenase (NAD(+))
KEGG Compound: C00022	Pyruvate	C00022	LCTONWCANYUPML-UHFFFAOYSA-N	1.2.1.49	2-oxoaldehyde dehydrogenase (NADP(+))

The list of enzymes retrieved using the above query can be used to query additional functional relationships that can help explain the significance of the metabolomics profiles being sampled.

Next, we demonstrate this feature using L-lactate-dehydrogenase as an example.

Step 5-A: Select "Enzyme" from the drop-down menu of data types and click the "Select" button.

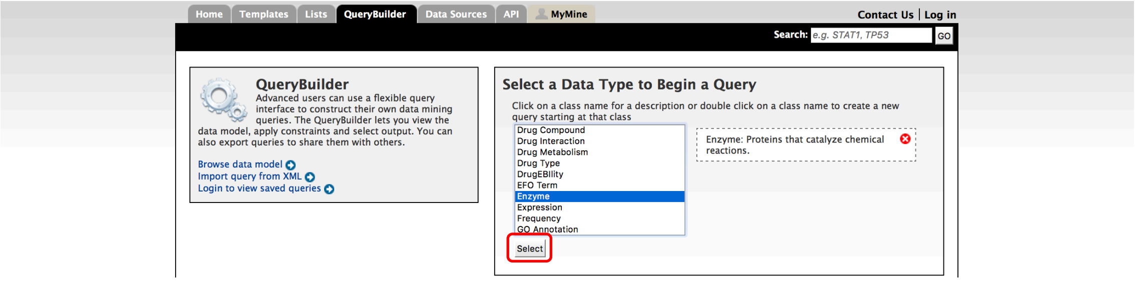

Step 5-B: In the model browser, select "Enzyme" and then and click the corresponding labels ("Description" and "EC Number") to select the desired attributes for output. To restrict the output results, we have constrained the query for 1.1.1.27 (L-lactate-dehydrogenase) using the "CONSTRAIN" function.

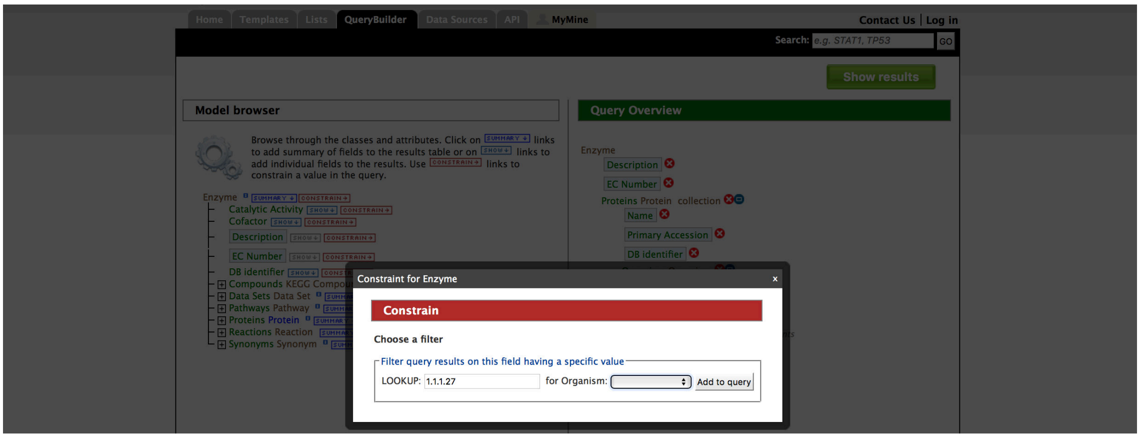

Step 5-C: Click "Show results" button to display the output.

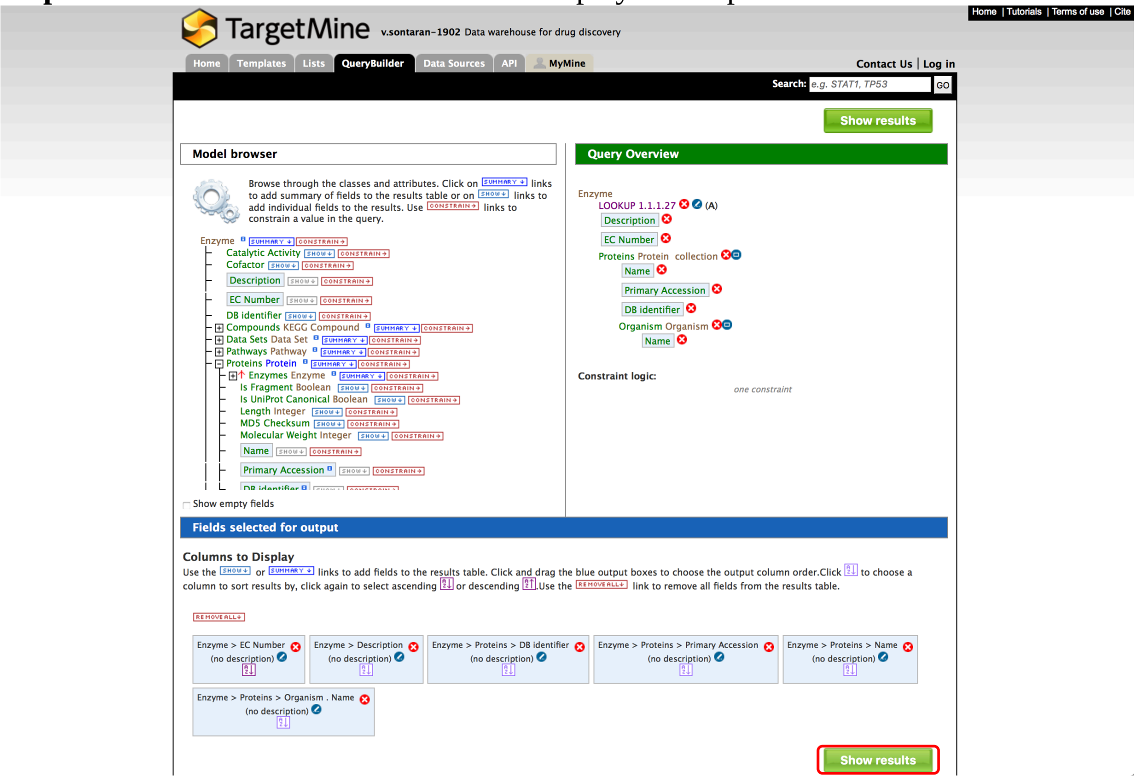

Step 5-D: The output table returns a list of proteins that encode for L-lactate-dehydrogenase and similar enzymes and their subunits in different species.

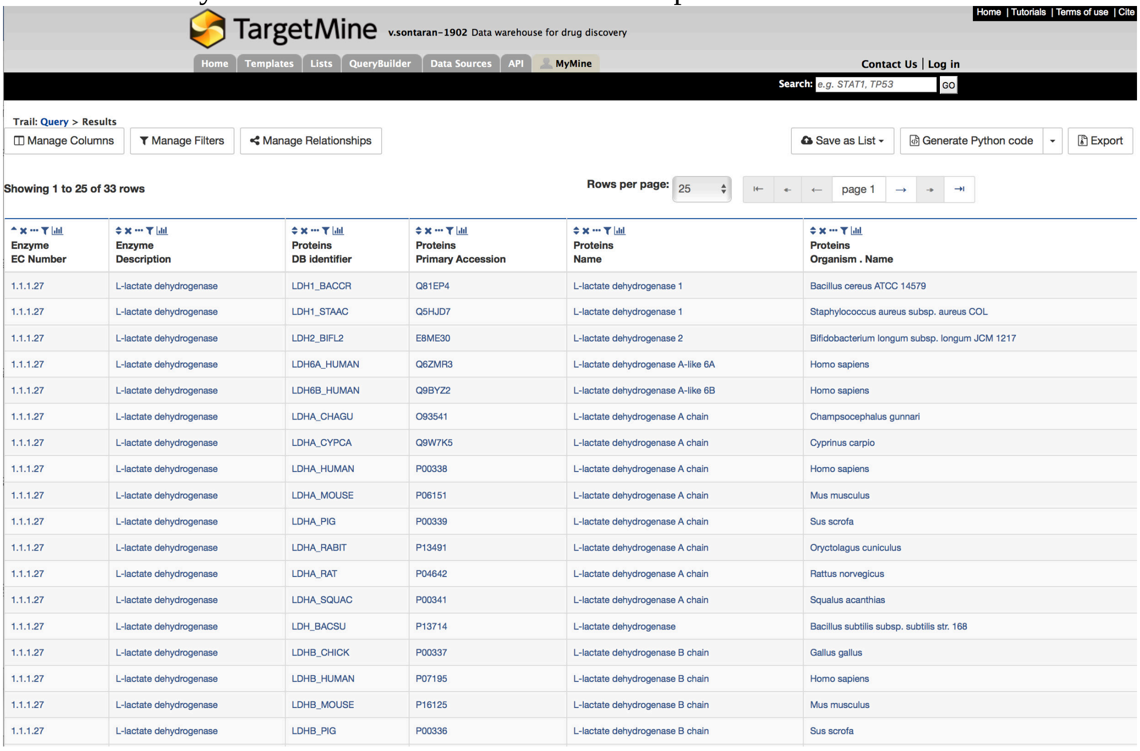

Enzyme EC Number	Enzyme Description	Proteins DB identifier	Proteins Primary Accession	Proteins Name	Proteins Organism . Name
1.1.1.27	L-lactate dehydrogenase	LDH1_BACCR	Q81EP4	L-lactate dehydrogenase 1	Bacillus cereus ATCC 14579
1.1.1.27	L-lactate dehydrogenase	LDH1_STAAC	Q5HJD7	L-lactate dehydrogenase 1	Staphylococcus aureus subsp. aureus COL
1.1.1.27	L-lactate dehydrogenase	LDH2_BIFL2	E8ME30	L-lactate dehydrogenase 2	Bifidobacterium longum subsp. longum JCM 1217
1.1.1.27	L-lactate dehydrogenase	LDH6A_HUMAN	Q6ZMR3	L-lactate dehydrogenase A-like 6A	Homo sapiens
1.1.1.27	L-lactate dehydrogenase	LDH6B_HUMAN	Q9BYZ2	L-lactate dehydrogenase A-like 6B	Homo sapiens
1.1.1.27	L-lactate dehydrogenase	LDHA_CHAGU	O93541	L-lactate dehydrogenase A chain	Champsocephalus gunnari
1.1.1.27	L-lactate dehydrogenase	LDHA_CYPCA	Q9W7K5	L-lactate dehydrogenase A chain	Cyprinus carpio
1.1.1.27	L-lactate dehydrogenase	LDHA_HUMAN	P00338	L-lactate dehydrogenase A chain	Homo sapiens
1.1.1.27	L-lactate dehydrogenase	LDHA_MOUSE	P06151	L-lactate dehydrogenase A chain	Mus musculus
1.1.1.27	L-lactate dehydrogenase	LDHA_PIG	P00339	L-lactate dehydrogenase A chain	Sus scrofa
1.1.1.27	L-lactate dehydrogenase	LDHA_RABIT	P13491	L-lactate dehydrogenase A chain	Oryctolagus cuniculus
1.1.1.27	L-lactate dehydrogenase	LDHA_RAT	P04642	L-lactate dehydrogenase A chain	Rattus norvegicus
1.1.1.27	L-lactate dehydrogenase	LDHA_SQUAC	P00341	L-lactate dehydrogenase A chain	Squalus acanthias
1.1.1.27	L-lactate dehydrogenase	LDH_BACSU	P13714	L-lactate dehydrogenase	Bacillus subtilis subsp. subtilis str. 168
1.1.1.27	L-lactate dehydrogenase	LDHB_CHICK	P00337	L-lactate dehydrogenase B chain	Gallus gallus
1.1.1.27	L-lactate dehydrogenase	LDHB_HUMAN	P07195	L-lactate dehydrogenase B chain	Homo sapiens
1.1.1.27	L-lactate dehydrogenase	LDHB_MOUSE	P16125	L-lactate dehydrogenase B chain	Mus musculus
1.1.1.27	L-lactate dehydrogenase	LDHB_PIG	P00336	L-lactate dehydrogenase B chain	Sus scrofa

Step 6-A: Select "Protein" from the drop-down menu of data types and click the "Select" button.

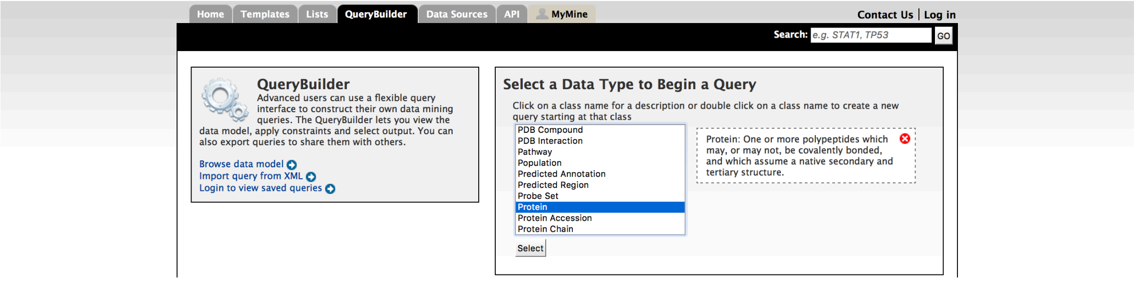

Step 6-B: In the model browser, select "Protein" and "Protein > Organism" and then click the corresponding labels (Protein- "Name", "DB identifier", "Symbol"; Organism- "Genus", "Name", Species", "Taxon Id") to select the desired attributes for output. To restrict the output results, we have constrained the query for P06151 (L-lactate-dehydrogenase A chain; Mus musculus) using the "CONSTRAIN" function.

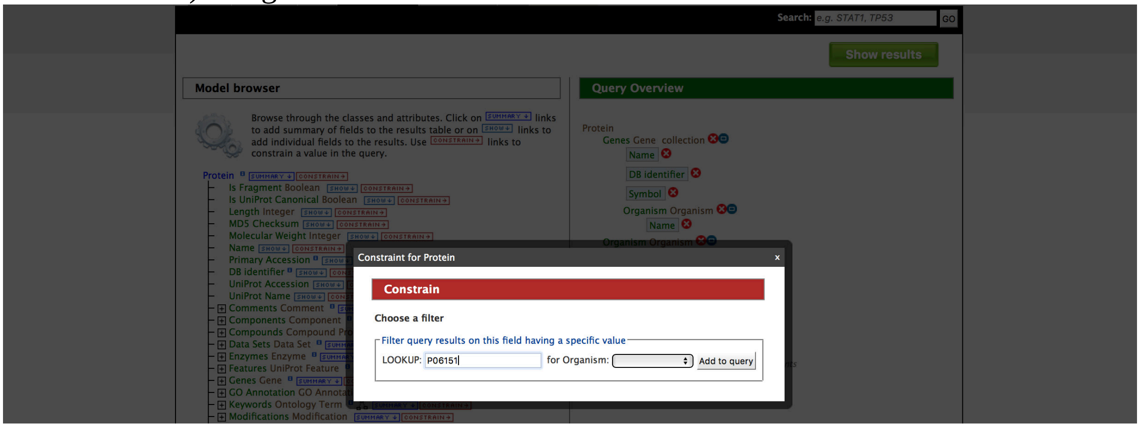

Step 6-C: Click "Show results" button to display the output.

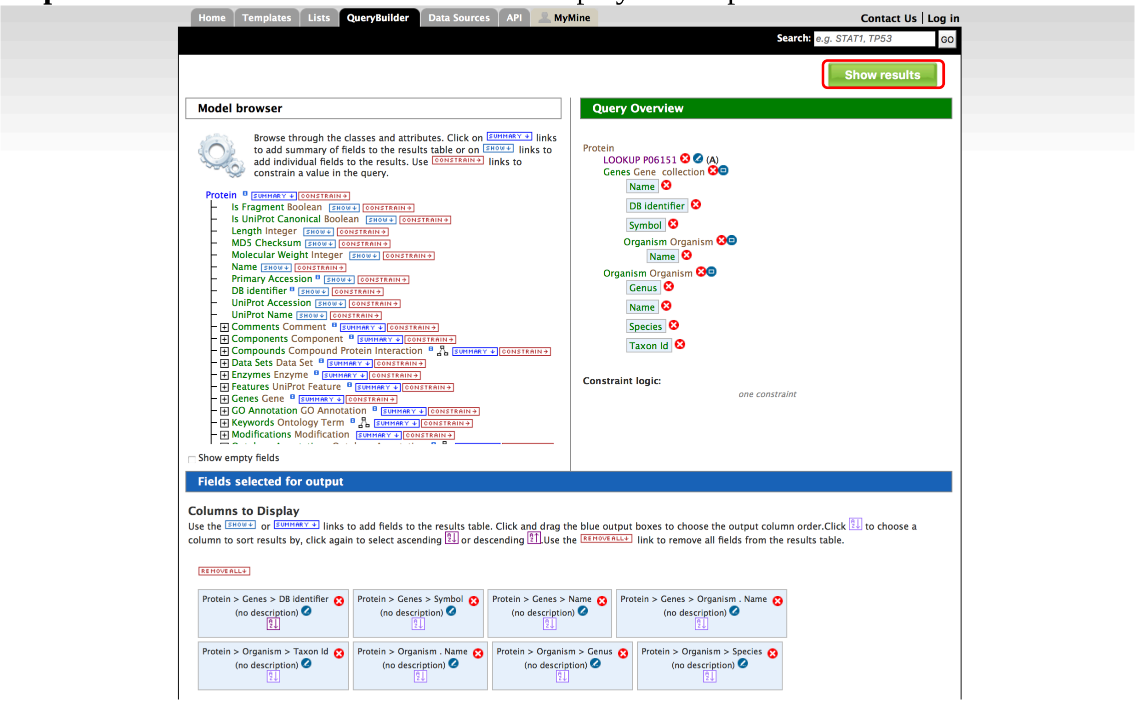

Step 6-D: The output table returns a list of identifiers associated with the gene that encodes for L-lactate-dehydrogenase A.

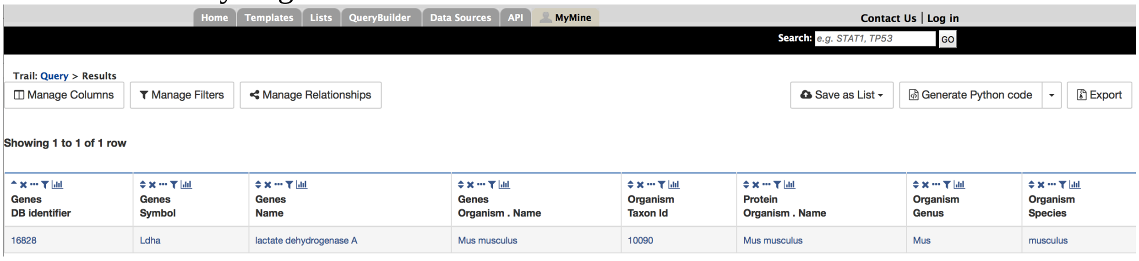

Next, we examine the tissue-specific expression of L-lactate-dehydrogenase A using the "Gene" class in TargetMine

Step 7-A: Select "Gene" from the drop-down menu of data types and click the "Select" button.

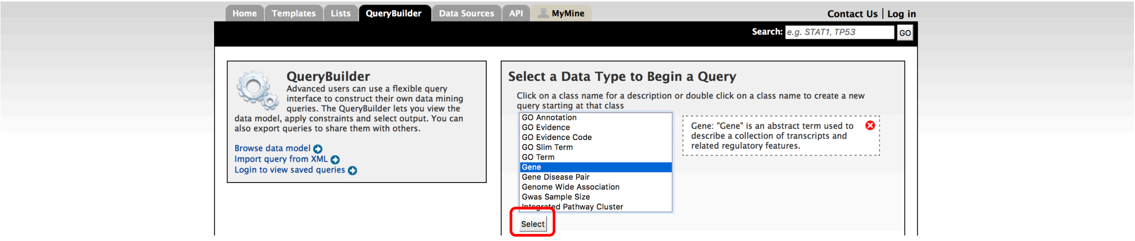

Step 7-B: In the model browser, select "Gene", "Gene > Probe Sets", "Gene > Probe Sets > Expressions", "Gene > Probe Sets > Expressions> Tissue" and "Gene > Probe Sets > Expressions > Organism" and then and click the corresponding labels. (Gene- "Name", "DB identifier", "Symbol"; Probe Sets- "Mapping Type", "Probe Set Id"; Expressions- "Value"; Tissue- "Identifier", "Name"; Organism- "Name") to select the desired attributes for output. To restrict the output results, we have constrained the query for 16828 (L-lactate-dehydrogenase A) using the "CONSTRAIN" function.

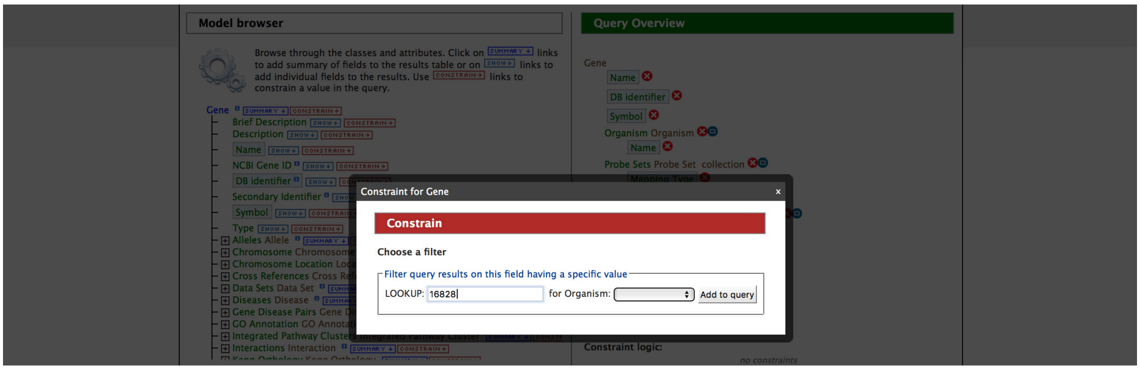

Step 7-C: Click “Show results” button to display the output.

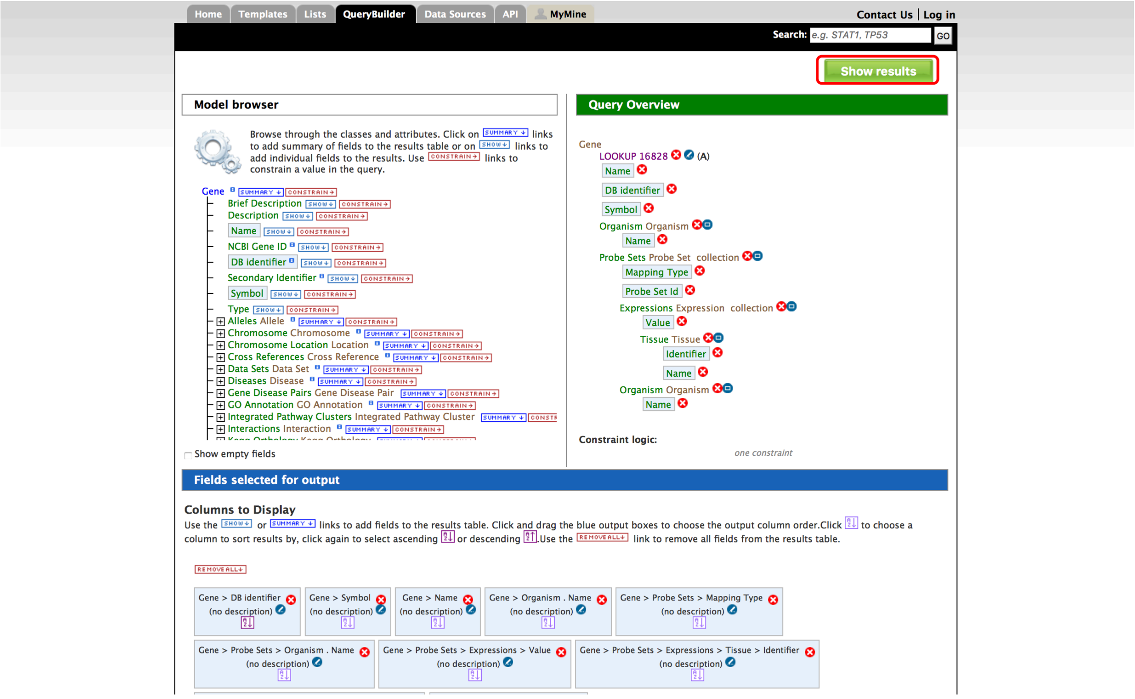

Step 7-D: The output table returns a list of tissues and the associated “Expressions value” of L-lactate-dehydrogenase A.

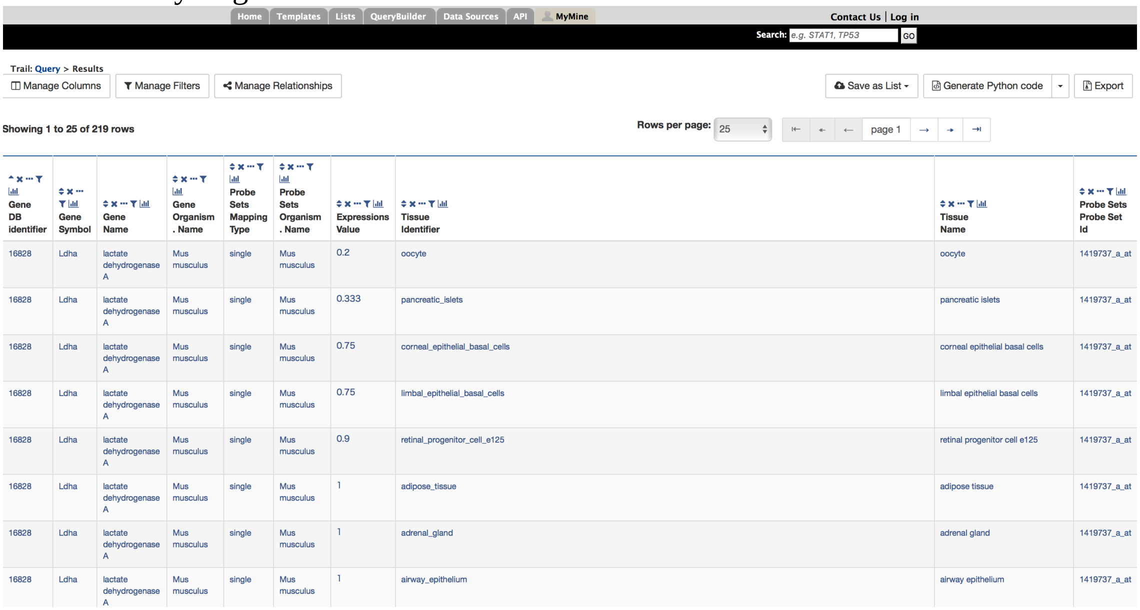

Gene DB identifier	Gene Symbol	Gene Name	Gene Organism . Name	Probe Sets Mapping Type	Probe Sets Organism . Name	Expressions Value	Tissue Identifier	Tissue Name	Probe Sets Probe Set Id
16828	Ldha	lactate dehydrogenase A	Mus musculus	single	Mus musculus	0.2	oocyte	oocyte	1419737_a_at
16828	Ldha	lactate dehydrogenase A	Mus musculus	single	Mus musculus	0.333	pancreatic_islets	pancreatic islets	1419737_a_at
16828	Ldha	lactate dehydrogenase A	Mus musculus	single	Mus musculus	0.75	corneal_epithelial_basal_cells	corneal epithelial basal cells	1419737_a_at
16828	Ldha	lactate dehydrogenase A	Mus musculus	single	Mus musculus	0.75	limbal_epithelial_basal_cells	limbal epithelial basal cells	1419737_a_at
16828	Ldha	lactate dehydrogenase A	Mus musculus	single	Mus musculus	0.9	retinal_progenitor_cell_e125	retinal progenitor cell e125	1419737_a_at
16828	Ldha	lactate dehydrogenase A	Mus musculus	single	Mus musculus	1	adipose_tissue	adipose tissue	1419737_a_at
16828	Ldha	lactate dehydrogenase A	Mus musculus	single	Mus musculus	1	adrenal_gland	adrenal gland	1419737_a_at
16828	Ldha	lactate dehydrogenase A	Mus musculus	single	Mus musculus	1	airway_epithelium	airway epithelium	1419737_a_at
