# Supplementary material for: The TargetMine Data Warehouse: Enhancement and Updates
Source: Front Genet. 2019 Oct 9;10:934. doi: 10.3389/fgene.2019.00934 (PMC6794636; doi:10.3389/fgene.2019.00934)
Supplement: Supplementary file 3 [file DataSheet_2.pdf]

Supplementary File S2: Extraction of gene-disease associations from literature using MeSH descriptors

Note: The users can click the "+" symbol to expand the class/node of interest and view the available nodes/classes within.

Step 1: Click the "QueryBuilder" tab in the tab panel

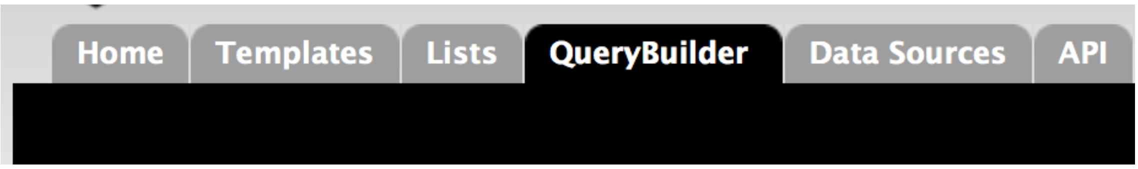

Step 2: Select "Gene" from the drop-down menu of data types and click the "Select" button.

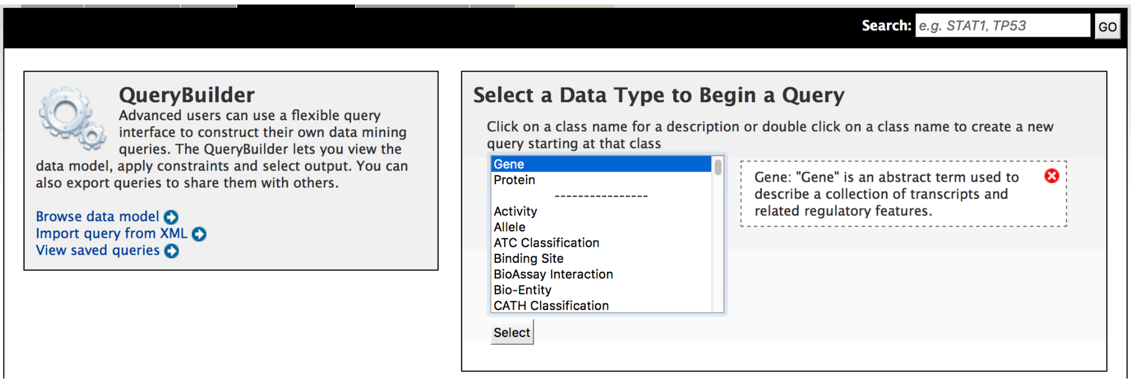

Step 3-A: In model browser, select "Publication>MeSH Terms" and click the "SUMMARY" label to display the default MeSH term attributes.

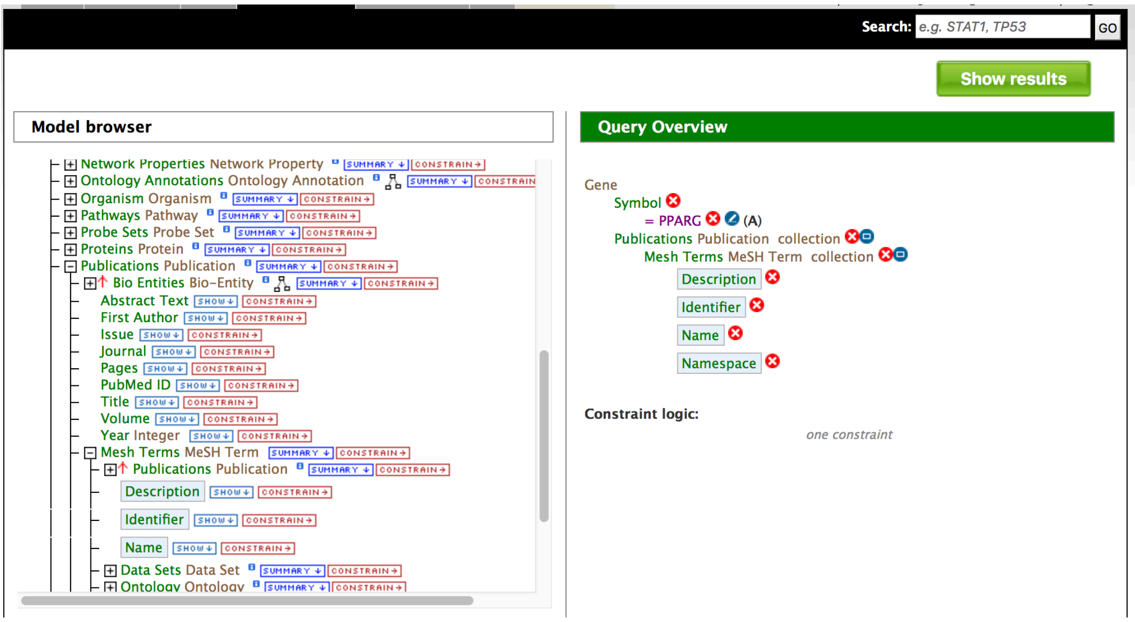

Step 3-B: To restrict the search to a specific gene (e.g. PPARG; Organism- H. sapiens), click the "CONSTRAIN" label next to "Symbol" tab and enter the gene symbol and click "Add to query".

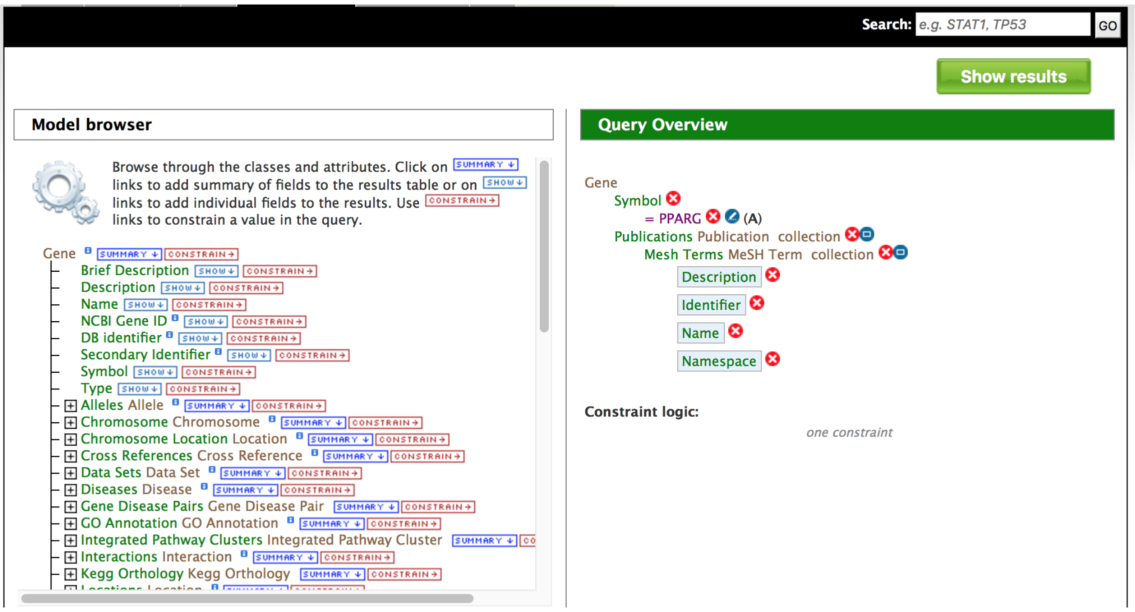

Constrain for gene (Gene Symbol; PPARG):

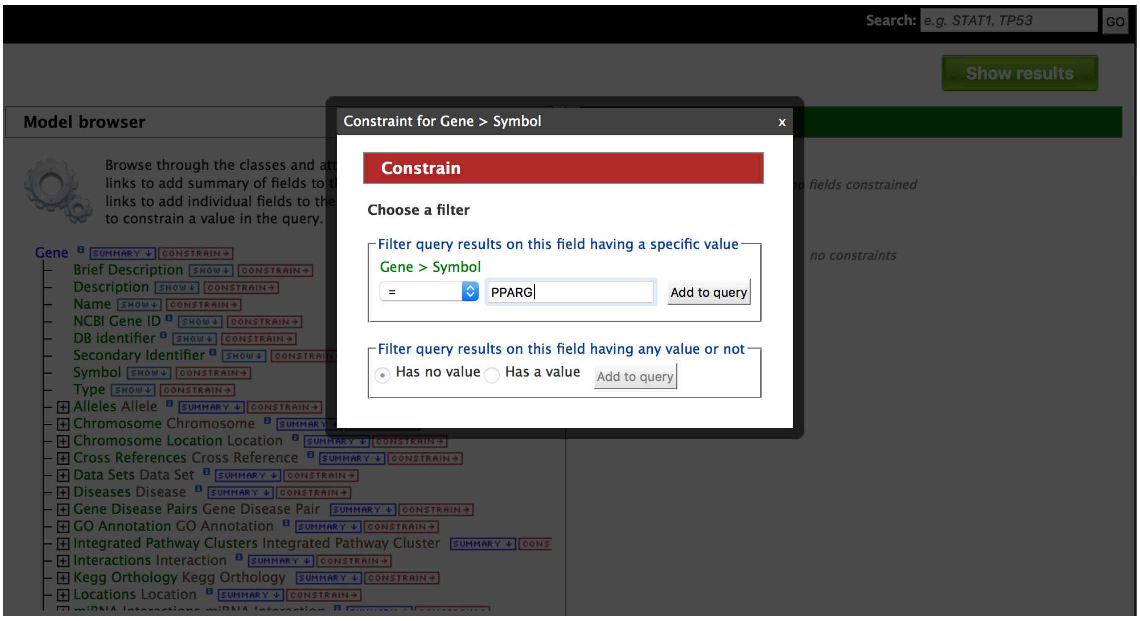

Constrain for organism (by Short Name- H. sapiens)

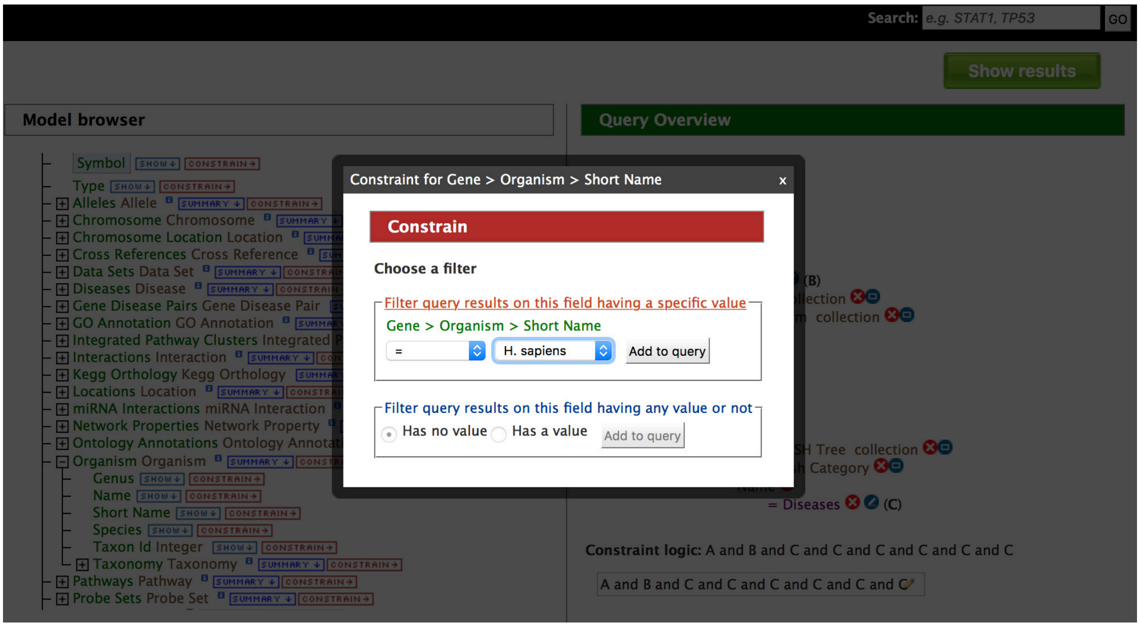

Step 4: To restrict the output to gene-disease associations, select “Publication->MeSH Terms->Tree Numbers->Category” and click the “CONSTRAIN” label next to “Name”. Select “Diseases” from the list of items in the drop-down menu and click “Add to query”.

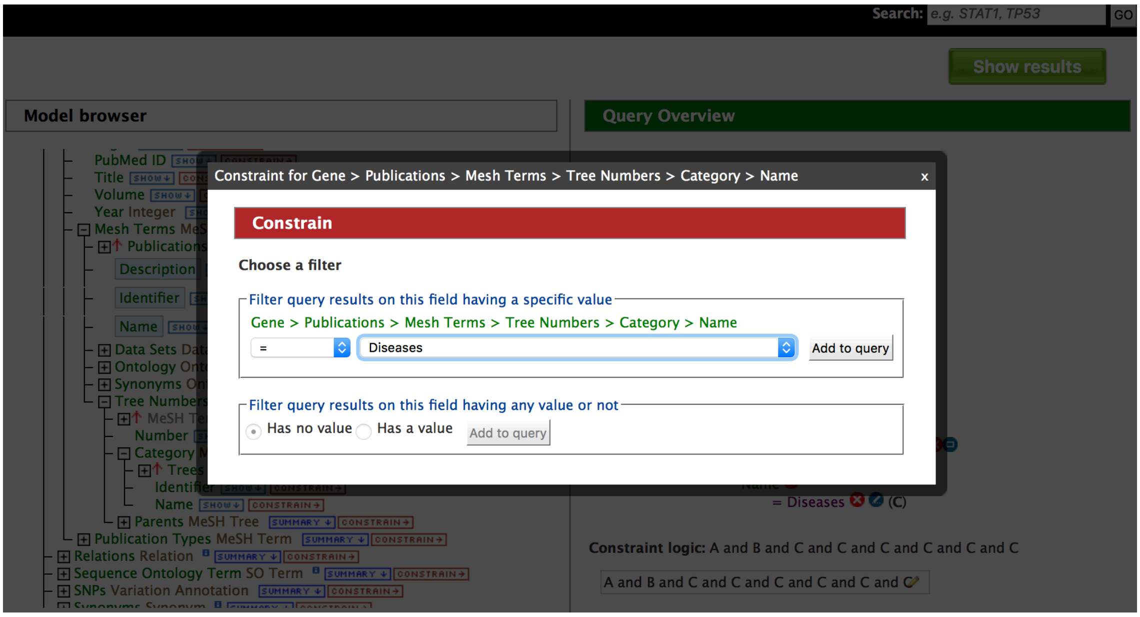

Step 5: Click the “Show results” button on upper right of the panel to view the output.

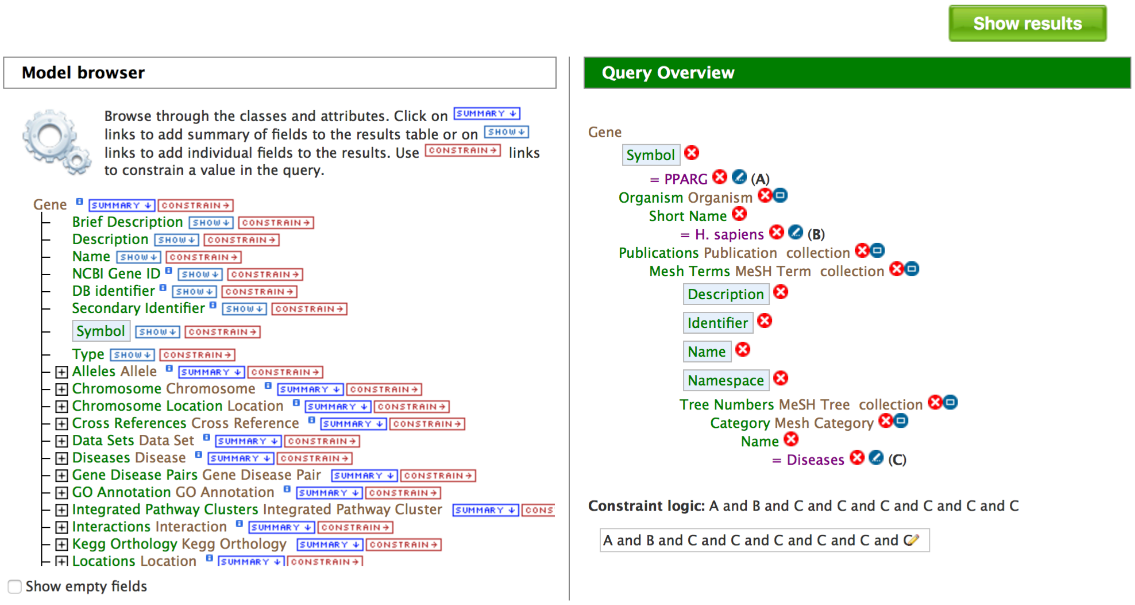

Results table:

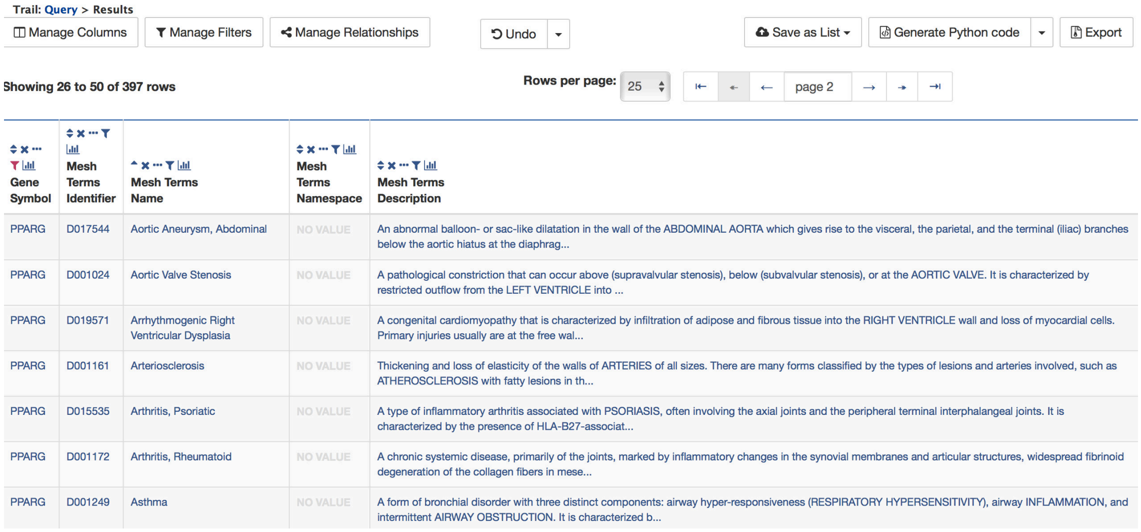

Gene Symbol	Mesh Terms Identifier	Mesh Terms Name	Mesh Terms Namespace	Mesh Terms Description
PPARG	D017544	Aortic Aneurysm, Abdominal	NO VALUE	An abnormal balloon- or sac-like dilatation in the wall of the ABDOMINAL AORTA which gives rise to the visceral, the parietal, and the terminal (iliac) branches below the aortic hiatus at the diaphrag...
PPARG	D001024	Aortic Valve Stenosis	NO VALUE	A pathological constriction that can occur above (supravalvular stenosis), below (subvalvular stenosis), or at the AORTIC VALVE. It is characterized by restricted outflow from the LEFT VENTRICLE into ...
PPARG	D019571	Arrhythmogenic Right Ventricular Dysplasia	NO VALUE	A congenital cardiomyopathy that is characterized by infiltration of adipose and fibrous tissue into the RIGHT VENTRICLE wall and loss of myocardial cells. Primary injuries usually are at the free wal...
PPARG	D001161	Arteriosclerosis	NO VALUE	Thickening and loss of elasticity of the walls of ARTERIES of all sizes. There are many forms classified by the types of lesions and arteries involved, such as ATHEROSCLEROSIS with fatty lesions in th...
PPARG	D015535	Arthritis, Psoriatic	NO VALUE	A type of inflammatory arthritis associated with PSORIASIS, often involving the axial joints and the peripheral terminal interphalangeal joints. It is characterized by the presence of HLA-B27-associat...
PPARG	D001172	Arthritis, Rheumatoid	NO VALUE	A chronic systemic disease, primarily of the joints, marked by inflammatory changes in the synovial membranes and articular structures, widespread fibrinoid degeneration of the collagen fibers in mese...
PPARG	D001249	Asthma	NO VALUE	A form of bronchial disorder with three distinct components: airway hyper-responsiveness (RESPIRATORY HYPERSENSITIVITY), airway INFLAMMATION, and intermittent AIRWAY OBSTRUCTION. It is characterized b...

Step 6: The users can export the results by “Export” button on the top-right.
