# Supplementary material for: The TargetMine Data Warehouse: Enhancement and Updates
Source: Front Genet. 2019 Oct 9;10:934. doi: 10.3389/fgene.2019.00934 (PMC6794636; doi:10.3389/fgene.2019.00934)
Supplement: Supplementary file 4 [file DataSheet_3.pdf]

Supplementary Figure 1: Trans-omics data analysis with TargetMine- usage example

Trans-omics gene set conversion, comparison and overlap

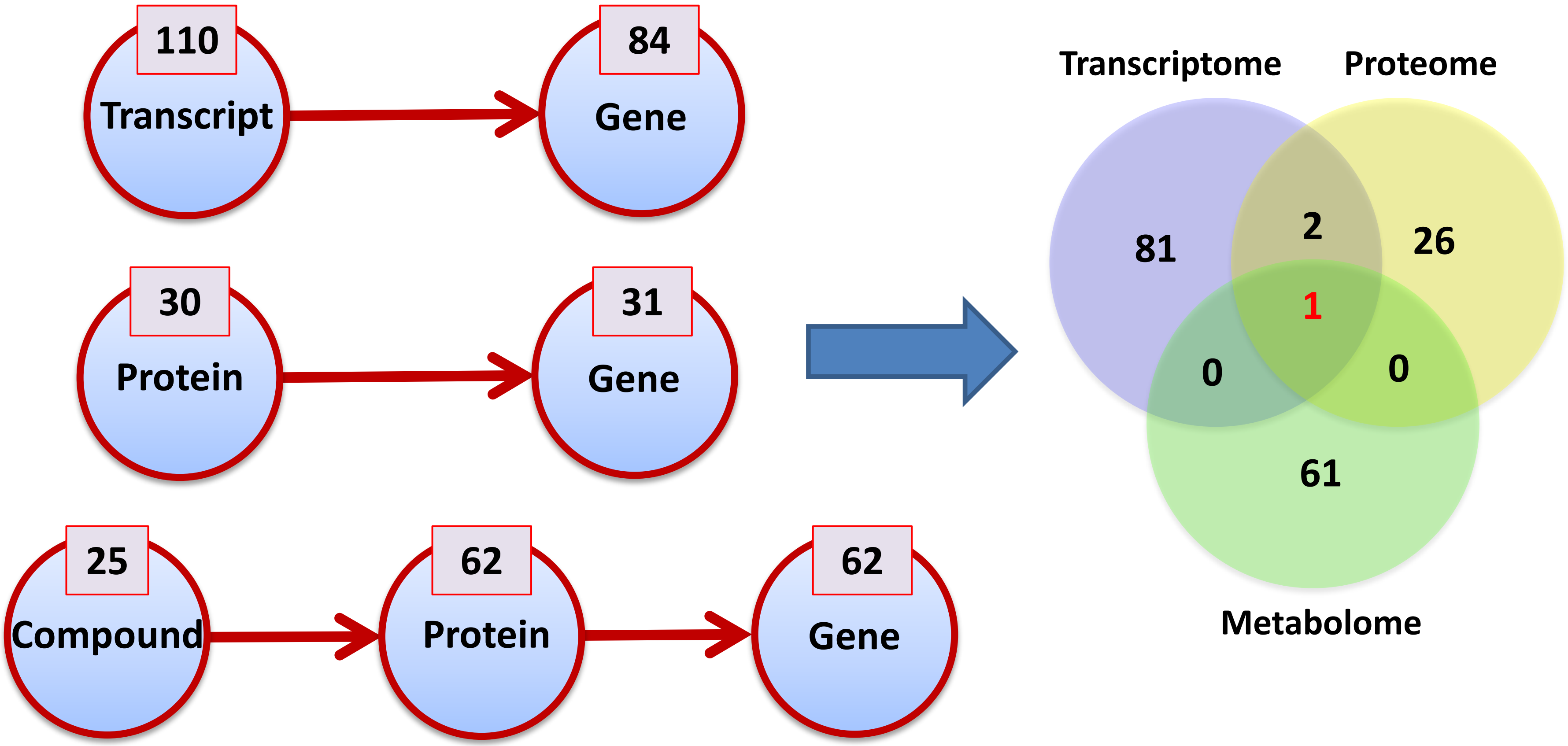

Gene Name	Gene Symbol	Transcript FC	Protein FC	Metabolite FC
carboxylesterase 2A	Ces2a	0.591	0.657	-
**cysteine sulfinic acid decarboxylase**	**Csad**	**0.544**	**0.666**	**0.643**
cytochrome P450, family 3, subfamily a, polypeptide 11	Cyp3a11	0.564	0.603	-

KEGG Pathway overlap- Subclass

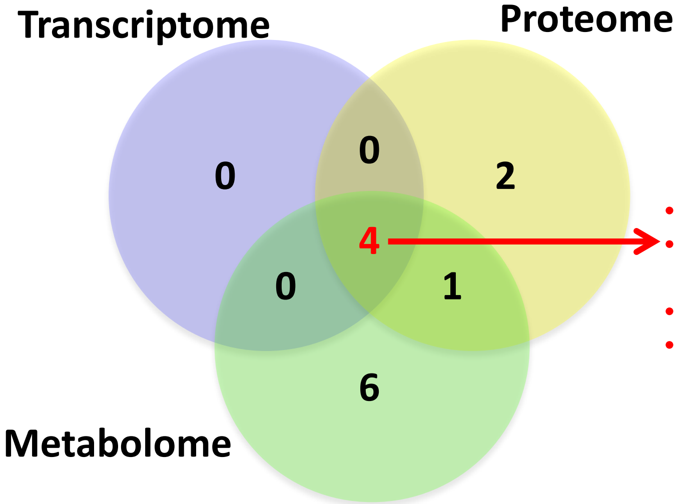

- Lipid Metabolism
- Global and overview maps
- Cancers: Overview
- Metabolism of cofactors and vitamins

KEGG Pathway overlap

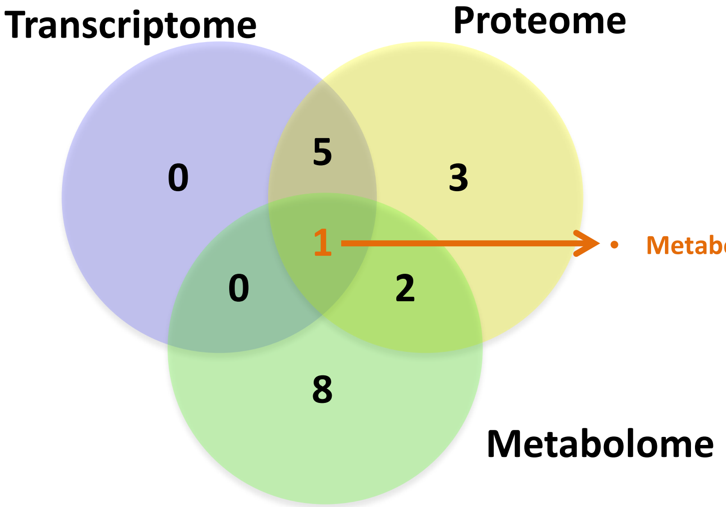

- Metabolic pathways

KEGG Pathway overlap and overlapping genes

KEGG Pathway	KEGG Pathway sub-class	Transcriptome	Proteome	Metabolome
Metabolic pathways	Global and overview maps	Csad	Csad	Csad
Linoleic acid metabolism	Lipid Metabolism	Cyp3a11	Cyp3a11	-
Retinol metabolism	Metabolism of cofactors and vitamins	Cyp3a11	Cyp3a11	-
Steroid hormone biosynthesis	Lipid Metabolism	Cyp3a11	Cyp3a11	-
Chemical carcinogenesis	Cancers: overview	Cyp3a11	Cyp3a11	-
Drug metabolism- other enzymes		-	5 genes (No gene overlap)	4 genes (No gene overlap)
